# On the Importance of Being Flexible: Dynamic Brain Networks and Their Potential Functional Significances

**DOI:** 10.3389/fnsys.2021.688424

**Published:** 2022-01-21

**Authors:** Adam Safron, Victoria Klimaj, Inês Hipólito

**Affiliations:** ^1^Center for Psychedelic and Consciousness Research, Department of Psychiatry and Behavioral Sciences, Johns Hopkins University School of Medicine, Baltimore, MD, United States; ^2^Kinsey Institute, Indiana University, Bloomington, IN, United States; ^3^Cognitive Science Program, Indiana University, Bloomington, IN, United States; ^4^Complex Networks and Systems, Informatics Department, Indiana University, Bloomington, IN, United States; ^5^Department of Philosophy, Berlin School of Mind and Brain, Humboldt-Universität zu Berlin, Berlin, Germany; ^6^Wellcome Centre for Human Neuroimaging, University College London, London, United Kingdom

**Keywords:** dynamical systems, flexibility, cohesion, criticality, brain entropy, free energy, Markov blankets, modules

## Abstract

In this theoretical review, we begin by discussing brains and minds from a dynamical systems perspective, and then go on to describe methods for characterizing the flexibility of dynamic networks. We discuss how varying degrees and kinds of flexibility may be adaptive (or maladaptive) in different contexts, specifically focusing on measures related to either more disjoint or cohesive dynamics. While disjointed flexibility may be useful for assessing neural entropy, cohesive flexibility may potentially serve as a proxy for self-organized criticality as a fundamental property enabling adaptive behavior in complex systems. Particular attention is given to recent studies in which flexibility methods have been used to investigate neurological and cognitive maturation, as well as the breakdown of conscious processing under varying levels of anesthesia. We further discuss how these findings and methods might be contextualized within the Free Energy Principle with respect to the fundamentals of brain organization and biological functioning more generally, and describe potential methodological advances from this paradigm. Finally, with relevance to computational psychiatry, we propose a research program for obtaining a better understanding of ways that dynamic networks may relate to different forms of psychological flexibility, which may be the single most important factor for ensuring human flourishing.

*“Mind thinks itself because it shares the nature of the object of thought; for it becomes an object of thought in coming into contact with and thinking its objects, so that mind and object of thought are the same.”*

*-Aristotle, Metaphysics*

*“We may thus lay it down as an established fact that the most perfected parts of the brain are those whose action are least determinate. It is this very vagueness which constitutes their advantage.”*

*-William James, Are we Automata?*

## Introduction

In what follows we consider dynamic perspectives on the brain and mind from methodological, neurological, and psychological views. The topics covered range from the highly intuitive to the highly technical. While we endeavored to provide sufficiently detailed handlings to provide understanding of important connections (please see Glossary), some parts will be highly challenging for people without background in those domains. However, readers can safely engage with these discussions in an “a la carte” manner, focusing on material of particular interest, with an ‘impressionistic’ understanding of technical portions being sufficient for following the overall trajectory of the paper through idea-space (Safron et al., 2021). While we believe there is a deep complementarity between these various points of view, we have constructed this paper with some degree of modular organization for the sake of efficient/flexible communication.

In what follows, this flexibility/modularity also applies to particular claims and theoretical commitments. For example, while at points a strong case is made for replacing standard models of representation-based cognition with “enactive” dynamical systems, these arguments are independent of (but potentially highly relevant to) the dynamic network methods we describe. Further, while we emphasize the dynamic point of view here, we also acknowledge that brains could be understood as hybrid ‘architectures’ with both representational and non-representational sub-systems (Friston et al., 2017a; Parr and Friston, 2018). [Alternatively, one could instead think of these quasi-representational elements of nervous systems as highly-connected causal structures that are particularly effective at bringing order to the overall mental economy/ecosystem]. By the end of these explorations, we hope to have provided an overview of some of the myriad ways in which dynamical perspectives may provide insights into the nature(s) of flexibility as a core organizing principle for understanding the cognitive abilities of brains, and perhaps complex adaptive systems more generally.

## Brains and Minds as Dynamical Systems

Finding meaningful patterns in neuronal activity is the primary basis of neuroimaging studies. A major challenge arises from the observation that interactions are highly context-specific. This means that neuronal events involve high degrees of freedom, thereby “living” in high dimensions and extremely hard to predict. The challenge in neuroimaging involves precisely estimating the unknown causes of neuronal activity (what we do not know) with neuroimaging data sets possessing many attributes (what we know). Thus, a common goal in neuroimaging is to develop and fit the model that best explains the causes of the observed data, i.e., neuronal activity.

One possible way of understanding and modeling neuronal activity is in terms of its hypothesized computational bases. This means that neuronal activity, seemingly, unfolds in the context of highly fixed structures such as domain-specific modules with highly specific information transmission properties (Arbib et al., 2003; Sporns and Betzel, 2016; Yan and Hricko, 2017; Damicelli et al., 2019; Mattar and Bassett, 2019). Even single neurons may be understood as modules, supposedly representing their respective parameters, e.g., state variables. In this neural network account, we attempt to obtain explanatory purchase by mapping out the topologies of functional connections maintained between modules.

Dynamical modeling has evolving dynamics at its target of explanation, rather than computational inferences based on static connectivity (Friston, 2011; Zarghami and Friston, 2020). This paradigm considers the brain as a dynamical system (as opposed to a modular, information processing computer).^1^ This dynamical renaissance in understanding nervous systems has been compellingly described by Favela (2020). Although the methods are not particularly new – e.g., differential equations – unfolding developments make these approaches central to the defense of the “dynamical hypothesis” in neuroscience. An account of the brain under the “dynamic hypothesis” explains neuronal activity using concepts such as “fixed point attractors,” “limit cycles,” and “phase transitions.” The concept of an attractor is particularly important, constituted by areas of state space moved toward by the trajectory of a system. High-dimensional systems like the brain tend to be computationally challenging to assess by virtue of having multiple phase transitions and attractors. Dynamical Systems Theory (DST) is uniquely qualified to deal with these high-dimensional processes characteristic of neurobiological systems. A dynamical system is an abstract description of physical identity, with a rule that can be specified at any given time by a set of variables. One possible example is as follows:


(1) $$\frac{d}{dt}x=f\left(x,t,u,\beta \right)+\omega$$

Such a system consists of a time derivative of of state of *x* as function of the present state of *x*, a controlled input (u), parameters (β) and stochastic forcing (ω), i.e., random influences that can only be modeled probabilistically. The function (f) is dynamic in the sense that it consists of a vector field with values at every point in space and time. This configuration allows us to predict future states by applying this dynamical rule for system evolution to the present state. Dynamical systems are specifically qualified to account for highly dimensional systems like the brain (with high degrees of freedom) by virtue of accounting for non-linearity.

Dynamical Systems Theory uses differential equations to describe system-evolution over time, where variables are treated as continuous, accounting for the highly situated, contextual nature of action in complex systems. An example is work on motor control through dissipative structures (Kugler et al., 1980), in which muscles involved in action are treated as coordinative structures of single units (see also Heylighen, 2016). This explanation is consistent with (1) Bernstein’s problem of how to explain the regulation of the many biokinematic degrees of freedom with minimal recourse to an “intelligent regulator” and (2) operation by the same organizational principles as other non-equilibrium thermodynamic systems. Importantly, this kind of work obviates needs for appealing to computational representations in many contexts.

Haken et al. (1985) offered a model of self-organization of perceptual-motor coordination such that different states are treated as dynamical patterns, as opposed to computations. A well-established (and of high instructional value) model in dynamical systems theory is Watt governors, which use mechanically-based negative feedback to regulate the amount of activity from steam engines. Such feedback mechanisms can also be used to explain aspects of cognitive activity without invoking computations or representations: “cognitive systems may in fact be dynamical systems, and cognition the behavior of some (non-computational) dynamical system” (Van Gelder, 1995, p. 358, italics in the original).

In this spirit, Hipólito et al. (2021a) recently advanced a model of action that does not require an optimal control system. Their model uses proprioceptive predictions to replace not only forward and inverse models with a generative model, but also obviates the need for motor plans (considered to be unrealistic due the required specificity of such plans and the many degrees of freedom of neuromuscular systems). Instead, perceptual-motor coordination is treated as coordinative structures of single units that operate by the same organizational principles as other non-equilibrium thermodynamic systems; more precisely, self-organized coordination in which different states are treated as dynamical patterns (without appealing to computational representations for explanatory power).

The “dynamical hypothesis” has also been applied to understanding brains as complex adaptive systems without appeal to representations or functional/computational principles. While there can be good reason to hold for pluralism in respect to combining structural and dynamical approaches for epistemic purposes, instrumental pluralism does not suffice to hold an ontology or ontological commitments. To put it more precisely, while it is possible that epistemic tools are combined to grant understanding, the ontological characterisation/understanding of neural activity differs between the computational/representational accounts (such as neural networks, deep learning, etc.), on the one hand, and the Dynamical Systems Theory, on the other. To encapsulate the traditional computation/representationalist account: “any system that is going to behave intelligently in the world must contain representations that reflect the structure of the world” (Poldrack, 2020, p. 1; see also Davis and Poldrack, 2013), holding the general assumption that “the mind is (1) an information processing system, (2) a representational device, and (3) (in some sense) a computer” (Bechtel and Graham, 1998, p. xiii). Dynamical Systems Theory, on the contrary, rejects the analogy of the mind/brain with a computer and the existence of neural representations altogether: “rather than computation, cognitive processes may be dynamical systems; rather than computation, cognitive processes may be state-space evolution within these very different kinds of systems” (Van Gelder, 1995, p. 346).

A notable example of this kind of dynamical handling is work in masses of neural sets in which cortical activity is explained *via* groups of action potentials (i.e., ‘masses’) that synchronize with other groups of neurons (Freeman, 1975; Freeman and Kozma, 2010). Another example is the model advanced by Hipólito et al. (2021b) in which neuronal circuits and organization do away with traditional, fixed modular processors. As will be described in greater detail below, this technique leverages the formalisms of Markov blankets (i.e., probabilistically-defined system boundaries based on conditional dependence/independence relationships) as well as active inference (i.e., a normative model of intelligent behavior) to analyze neuronal dynamics at multiple scales, ranging from single neurons, to brain regions, to brain-wide networks (Friston et al., 2021). This treatment is based upon canonical micro-circuitry characterized in empirical studies of dynamic effective connectivity, with potentially far-reaching practical applications for neuroimaging. This connection between macro- and meso-scale dynamics with microscale processes is especially relevant when considered within the framework of variational Bayes and information geometry (Ramstead et al., 2020), with further quantitative support obtainable *via* mathematical formalisms from Renormalisation Group theory (Friston et al., 2021) (we will return to this in the last section).

Zarghami and Friston (2020) developed a model of neural dynamics that generates trajectories in the parametric space of effective connectivity modes (i.e., states of connectivity). This approach allows for more detailed characterization of functional brain architectures by extending the powerful technique of spectral Dynamic Causal Modelling (DCM) (Friston et al., 2003, 2019; Daunizeau et al., 2011; Razi and Friston, 2016). Effective connectivity is – by definition – model-based. It develops hypothesis-driven generative models to explain empirical observations of neuronal activity (Friston, 2011). The effective connectivity paradigm is grounded in a view of brains in which continuously-expressed patterns of transiently coordinated activity emerge and dissolve in response to internal and external perturbations. Importantly, the emergence and evolution of such metastable coordination dynamics (in self-organizing complex systems, such as the brain) is inherently non-linear, context-sensitive, and thereby flexibly adaptable. It is through this dynamical perspective that we interpret the mesoscale neural phenomena described below (see also Atasoy et al., 2019; Wens et al., 2019).

The goal of generative modeling of neural phenomena is to explain how the brain generates neuroimaging data. This approach begins from the assumption that neuronal dynamics are generated by patterns of intrinsic (within region) and extrinsic (between region) connectivity, which continuously change through time. The generative modeling of these iterant brain states is motivated by empirical studies of dynamic functional connectivity (Vidaurre et al., 2017), theoretically-grounded in models in which macroscopic (slowly-evolving) dynamical modes (Haken, 1983; Jirsa et al., 1994) visit a succession of unstable fixed points in a parameter space for directed connectivity.

This technique enables the attainment of the main component for developing a dynamic model: its parameters configured according to a Markov process. This specification determines how connectivity parameter space will visit a succession of unstable fixed points (Rabinovich et al., 2008, 2012). Any natural system can be described as a dynamical system. By defining an initial condition and applying a dynamical rule in several iterations we obtain a set of trajectories that allow us to see the behavior of a system as approaching or avoiding certain points. This means that the behavior of a system can be seen as tending to visit stable fixed points and avoiding other points called repellers. For example, in a moving pendulum a repeller is a point at which the pendulum would remain upward. Pendulums do not tend to stay upward as they are attracted to a fixed, stable point, which is their resting state in a downward direction. We can thereby say that the behavior of a pendulum can be explained as moving against the repeller point (upward) and toward a stable, fixed point (downward). This can be plotted in an orbit or trajectory of a cycle between two points (between repeller and attractor points).

Zarghami and Friston (2020), in their generative model of neural connectivity, have two assumptions as their point of departure. First, connectivity patterns are assumed to trace out a heterocyclic orbit. A heterocyclic orbit is a kind of cycle, in which as time evolves, a typical trajectory would stay for increasingly longer period of time near a solution, which can be an equilibrium point, a periodic orbit, or a chaotic invariant set (in the case of effective connectivity below it is equilibria). Notably, an interesting property of heterocyclic cycles is that they are robust under perturbations (external force applied to the system’s activity).

**FIGURE 1:**
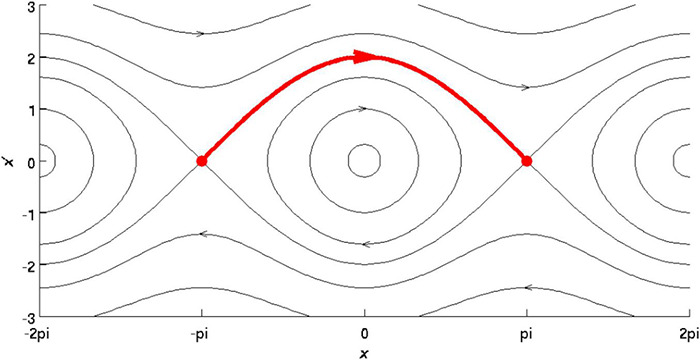
Reprinted from Wikipedia (CC): A SHC is a set in the phase space of a dynamical system that consists of a circle of equilibrium points and connecting heterocyclic connections. In this image, the *Y*-axis indicates varying trajectories, and the *X*-axis indicates phase with respect to particular orbits, with the red arrow indicating the completion of one revolution.

A Stable Heteroclinic Cycle (SHC) (Figure 1) describes the activity of the brain as trajectories visiting a discrete number of unstable fixed points in a winnerless competition among brain activity states (Afraimovich et al., 2008; Deco and Jirsa, 2012; Friston et al., 2014). Supposing neural activity as a heterocyclic cycle means that it is asymptomatically stable, i.e., can be described as approaching trajectories spend longer periods of time in a neighborhood of successful equilibria.

The second assumption is that transitions from one unstable fixed point to the next are relatively fast with respect to the duration over which effective connectivity remains in the neighburhood of a fixed-point attractor. This assumption leverages two further features of the SHC: (i) the origination of structural stability through self-organization, and (ii) long passages of time spent in the vicinity of saddles^2^ in the presence of moderate noise with high-dimensionality connectivity (with large numbers of degrees of freedom) (Rabinovich and Varona, 2018). In short, the two assumptions in the light of thinking effective connectivity as heteroclinic events are that wondering sets will (a) increasingly spend more time in stable points and (b) transitions from unstable point to another are fast.

Zarghami and Friston (2020) concluded that small random perturbations are unlikely to alter the global structure of heteroclinic events (recall that heterocyclic cycles is that they are robust under perturbations), but rather renders greater stochasticity in the duration of these events. This is consistent with evidence from EEG microstates as short time-periods of stable scalp potential fields (Michel and Koenig, 2018). Further, in agreement with (Stone and Holmes, 1990), while weak additive noise does not essentially alter the structure of phase space solutions, it induces radical change in leading to “a selection of timescales” in the evolution of dynamic interactions and their emergent/synergistic properties. Effective connectivity timescales might thereby be useful in informing canonical models of metastability for both normally functioning and pathological brains. In turn, the study of such diverse nervous systems and their associated properties can further elucidate the functional role of noise and manifold instabilities in inducing potentially altered heteroclinic structures and time-scales. This means that mapping the patterns of activity of effective connectivity would allow us to indicate patterns typically associated with well-adjusted or healthy neuropsychology, and, conversely, patterns associated with psychopathology. This is relevant because it takes us one step further from the topological structures of the brain typically mapped out by functional connectivity. Many functional principles cannot be reduced to particular neural structures, but instead require focusing on emergent patterns of activity that both influence and are influenced by multiple systems and their various combinations of dynamic interactions. An example of this is, with respect to our discussion (below) of neural systems enabling flexible adaptation, that many of these areas have been found to have less myelinated internal connectivity, potentially affording more slowly evolving dynamics as they integrate and influence other systems with which they couple (Haueis, 2021).

Although other paradigms, e.g., neural networks (Koch, 1999; Smith et al., 2020), note the importance of time-varying processes in neuronal activity, the question is the extent to which they depart from views in which brains are fundamentally understood as dynamical systems. As we have seen, the “dynamical hypothesis” attempts to explain neuronal activity in terms of concepts such as “fixed point attractors,” “limit cycles,” “phase transitions.” These conceptualizations and methodological approaches are what make dynamical systems theory uniquely qualified to explain the non-linear and context-sensitive evolution of neurobiological systems. Below we will explore particular analysis techniques that may be particularly apt for describing the flexibly adaptive character of brains as dynamical systems.

## Flexibility in Brains and Minds

How do functional aspects of brains emerge from the network properties of nervous systems? How do these functions and processes vary across and within individuals? Do greater tendencies toward exploration in behavior and cognition correlate with individuals having brains with greater (or more dynamic) degrees of interconnectivity (Beaty et al., 2016; Kenett et al., 2018)? Do more creative cognitive styles correlate with greater capacities for flexibly transitioning between metastable functional networks (Váša et al., 2015; Wens et al., 2019)? Could broader ranges of preferences or behaviors correspond to having a greater diversity of dynamics in functional networks (Beaty et al., 2015; Atasoy et al., 2017; Huang et al., 2020)? In which ways does the dynamic character of brain connectivity influence the development of people, personalities, and their capacities for ongoing learning/evolution through time and experience?

Here we attempt to describe how these questions might be addressed with methods for characterizing the dynamic properties of brains. We mostly focus on a measure of “network flexibility” as introduced by Bassett et al. (2011), which assesses the degree to which brain networks dynamically reconfigure themselves over time. To calculate network flexibility, the community structure of a brain is estimated at successive time windows in a measurement session. The degree to which nodes change their community allegiance across these time windows corresponds to network flexibility. Intuitively, brains in which nodes change their communities more often have greater overall flexibility. Similarly, individual nodes or groups of nodes can be assessed with respect to the frequency with which they change community allegiances. In this way, we can look at flexibility on multiple scales, so characterizing both global and regional dynamics (which may differ substantially), as well as their inter-relations.

The basic technique for estimating network flexibility involves dividing up a time series into a series of epochs, where the number of divisions depends on the sensitivity of the measure and estimated temporal granularity for phenomena of interest. Across these epochs, data is clustered into groups of correlated nodes known as cliques, or communities, or modules, which are assumed to reflect functional subnetworks over which relatively higher degrees of communication takes place. The modeler then determines the proportion of times that nodes switch community-allegiances, providing a number between 0 and 1, where 0 reflects minimally flexible nodes that never change cliques, and 1 reflects maximally flexible nodes that always change cliques. These values can then be averaged over the whole system to determine the entire network’s flexibility. Although in this paper we will focus on neuroscience applications, one of the many exciting things about this technique is that it can be applied to any time series data with a graph structure, which means any time-varying dataset.

In Bassett et al. (2011), overall brain network flexibility was found to predict the rate at which sequences were learned. In another study, flexibility in the striatum correlated with enhanced reinforcement learning of visual cues and outcomes (Gerraty et al., 2018). Whole-brain flexibility has been further shown to correlate with working memory as assessed by N-back tasks (Braun et al., 2015). An additional study replicated these N-back findings, and found correlations between whole-brain flexibility and number of hours slept, as well as performance on a relational reasoning and planning task (Pedersen et al., 2018). These findings may be further consistent with a study in which variability in flexibility was explained by fatigue (Betzel et al., 2017).

Betzel et al. (2017) also found that positive mood was correlated with reduced flexibility in the dorsal attention network (DAN), which is notable in highlighting that flexibility is not necessarily something that is strictly beneficial in all contexts. For example, Chai et al. (2016) found that language comprehension involved a relatively stable set of regions in the left hemisphere, but with a more flexible periphery of right-lateralized regions. As will be discussed in greater detail below, adaptive functioning may involve a kind of synergistic division of labor where systems are tuned to exhibit varying degrees of flexible responsiveness, which might reflect optimization for particular functional roles. Fascinatingly, this sort of “divide-and-conquer” approach to achieving both stability and plasticity may not only apply to networks of effective connectivity within brains, but also to emergent dynamic modules in the metabolism of individual cells (Jaeger and Monk, 2021).

Along these lines, a recent study found that excessive flexibility in the visual areas of infants negatively correlated with the rate at which subsequent developmental milestones were surpassed (Yin et al., 2020). Such a pattern might be expected if efficient perception is reflected by more stable intra-module dynamics. However, progressing through developmental stages was generally characterized by increased flexibility in somatomotor areas as well as higher-order brain regions including the temporoparietal junction, anterior cingulate, and anterior insula. This “flexible club” was largely distinct (60% non-overlap) from areas identified as functional hubs with high betweenness centrality, and also distinct from a “diverse club” of areas with high participation coefficients. These more flexible areas were also characterized by relatively weak (but more variable) connection strengths, which was suggested to “enable the system to function within many difficult-to-reach states, reflecting a capacity to adapt to novel situations.” Interestingly, flexibility in these areas has also been found to correlate with intelligence-related phenomena such as “need for cognition” and creative achievement (He et al., 2019). These flexible areas appear to constitute a combination of hubs from the default mode network involved in creative and imaginative cognition (Hassabis and Maguire, 2009) and ‘modeling’ of self and other (Saxe et al., 2006; Davey and Harrison, 2018), frontoparietal control/attention network (Huang et al., 2020), as well as salience-determining network for high-level action selection (Rueter et al., 2018; Toschi et al., 2018). These areas appear to facilitate overall metastability in the brain (Wens et al., 2019), with analogous mechanisms being observable across a potentially surprising range of organic systems (Hanson, 2021).

**FIGURE 2:**
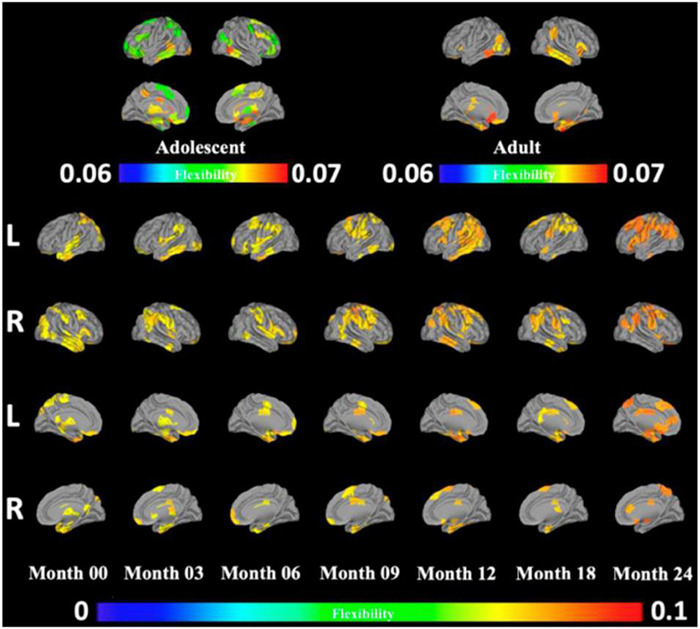
Reprinted with permissions from Yin et al. (2020). The development of the flexible club infancy, in adolescence, and in adulthood. Red indicates regions with significantly higher flexibility than the whole brain, and blue indicates regions with significantly lower flexibility than the whole brain. Orange indicates regions with no significant difference in flexibility from the whole brain.

While this “flexible club” was largely similar across age groups ranging from infants to adults, there were also notable differences (Yin et al., 2020) (Figure 2). From 0 to 24 months of age, infants gained flexibility in frontal and premotor areas, consistent with the gradual emergence of intentional control processes. Further, in moving from adolescence to adulthood, while frontal and somatomotor areas are included in the adolescent flexible club, these areas lose their relative flexibility in adulthood, consistent with adolescence being a time of intense change. The precuneus showed a more complex pattern of decreasing flexibility up to 3 months of age, followed by subsequent increases with further development. These findings are intriguing in light of the roles of this brain area in conscious visual perception and mental imagery (Utevsky et al., 2014; Ye et al., 2018; Safron, 2020). Perhaps even more intriguingly, the precuneus became relatively less flexible in adulthood, which could (speculatively) be taken to reflect a (potentially adaptive) reduction of psychological flexibility with age.

## Different Forms of Flexibility: Disjointedness vs. Cohesiveness

**FIGURE 3:**
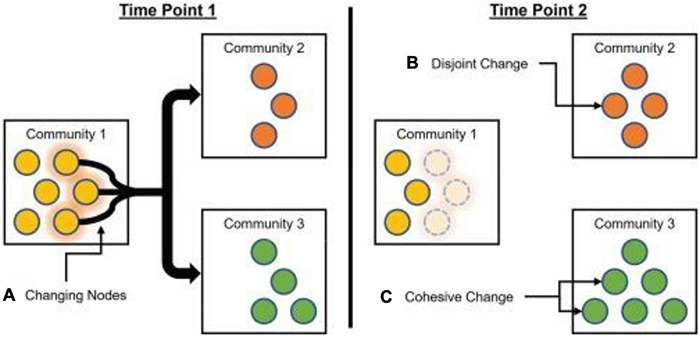
Schematic of cohesive and disjoint community changes. From Time Point 1 to Time Point 2, one yellow node from Community 1 **(A)** moves on its own to Community 2 **(B)**. This represents a disjoint change, with the node moving independently of other nodes. At the same time, two yellow nodes move from Community 1 **(A)** to Community 3 **(C)**, as a group. This represents a cohesive change, with nodes moving in tandem with each other. Reprinted with permission from Telesford et al. (2017).

Network flexibility is not necessarily strictly beneficial, and may exhibit an inverted U relationship with desirable characteristics (Yerkes and Dodson, 1908; Northoff and Tumati, 2019). Relatively elevated brain network flexibility may be associated with adaptive behaviors, but excessive flexibility may be indicative of systems pushed past an adaptive point of metastability (Carhart-Harris et al., 2014; Atasoy et al., 2019). For instance, flexibility has been shown to be elevated in people with schizophrenia, as well as in their relatives who may also be at increased risks for psychosis (Braun et al., 2016). However, Telesford et al. (2017) proposed two different variants of flexibility analyses with very different properties: disjoint flexibility measures the degree to which nodes tend to migrate separately; cohesive flexibility measures the degree to which nodes migrate together (Figure 3).

Node disjointedness is calculated by assessing the number of times a node changes communities independently, divided by the number of times a node could have potentially changed communities. Node cohesion is calculated by assessing the proportions of times a node changes communities in conjunction with other nodes in its (previous) community allegiance, summing over all pairwise co-migrations. For example, if a community splits into two new communities that each contain multiple nodes, the average disjointedness will be zero because all nodes migrate as part of a group, but average cohesion will be greater than zero (because each node migrates with at least some other nodes).

**FIGURE 4:**
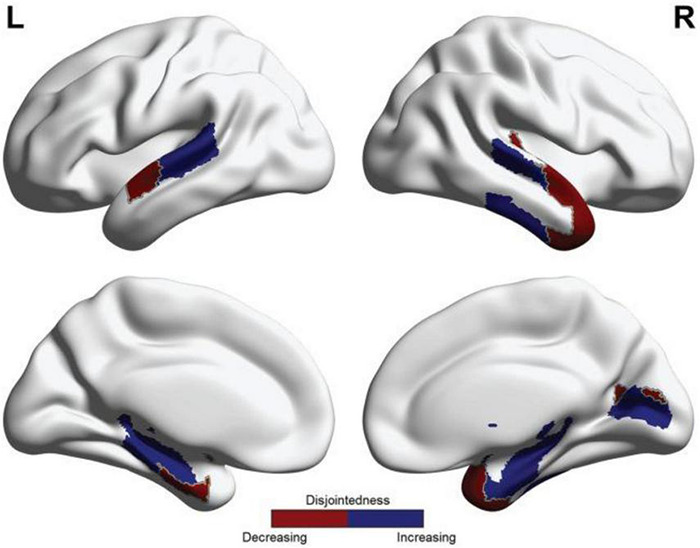
Reprinted with permissions from (Telesford et al., 2017). Patterns of changing disjoint flexibility as associated with learning. Blue indicates regions where disjointedness increased between Day 1 and Day 2 of the motor learning task, and red indicates regions where disjointedness decreased between Day 1 and Day 2 of the motor learning task.

While cohesive flexibility positively correlated with learning rate in Telesford et al. (2017), disjoint flexibility did not show this association, and was speculated to reflect something like a general measure of neural entropy. However, this should not be taken to indicate that disjoint dynamics are necessarily maladaptive. In this particular study (Figure 4), while hierarchically higher areas became less disjoint with training, hierarchically lower areas tended to become more disjoint. Speculatively, this pattern could be taken as indicating predictive processing in which higher-level coherent neural activity successfully suppresses bottom-up information flows (Bastos et al., 2020; Walsh et al., 2020).

While disjoint flexibility could be viewed as a neural entropy measure in terms of independent node switching, entropy has also been associated with adaptive processing and even intelligence (Carhart-Harris et al., 2014; Chang et al., 2018; Herzog et al., 2020; Vivot et al., 2020; Cieri et al., 2021). Thus, disjointedness may also sometimes exhibit an inverted U relationship with psychological functioning. Qualitatively speaking, low-to-moderate levels of disjointed migration may be better than stasis/rigidity, but the non-coherent nature of node migration may tend to indicate a breakdown of adaptive functioning. That is, it is possible that some degree of disjoint dynamics may be required for adaptability, but correspond to non-adaptive dynamics past a certain (and potentially low) threshold for stochasticity. A minimal degree of disjoint flexibility may correspond to adaptive meta-stability, and possibly regimes characterized by “stochastic resonance” (Vázquez-Rodríguez et al., 2017), which have been shown to have many desirable properties such as enhanced abilities to transmit and integrate information. However, excessive disjointedness could potentially indicate a disruption of integrated functioning of the kind associated with disconnection-type psychotic states (Friston et al., 2016). Cohesive flexibility, in contrast, might involve a more linear relationship with adaptive functioning, but with potentially maladaptive consequences at extremes (e.g., manic psychosis).

## Cohesive Flexibility, Criticality, and Consciousness?

Beyond its implications for psychological functioning, it may be the case that cohesive flexibility represents a hallmark of kinds of universality classes, or emergent properties that can be found across a wide range of complex systems. Along these lines, flexibility analyses may potentially be used as proxies for self-organized criticality (Bak et al., 1987; Atasoy et al., 2019). Self-organized criticality refers to the tendency of systems to exhibit phase transitions as attracting states. Not only does this self-organization allow for enhanced access to wider regions of phase space, but these “edge of chaos” inter-regimes also balance disordered and ordered dynamics, with sufficient variation to support adaptations, while also providing sufficient stability for the accumulation of structure in (generalized) evolution (Sneppen et al., 1995; Paperin et al., 2011). Such near-critical organization is also essential for inference/learning (Friston et al., 2012; Hoffmann and Payton, 2018), which can itself be considered to be a kind of evolutionary process (Campbell, 2016). These kinds of flexibly adaptive processes may also be essential for realizing consciousness as a dynamic core and integrated world model (Safron, 2020), whose emergent functioning further enhances the ability of systems to flexibly adapt to novel situations. This dynamical systems interpretation of the sources of flexibility could potentially be tested by looking for correlations with putative hallmarks of self-organized criticality such as power-law distributions, increased fractal dimension, and critical slowing down (Friston et al., 2012; Touboul and Destexhe, 2017; Varley et al., 2019).

**FIGURE 5:**
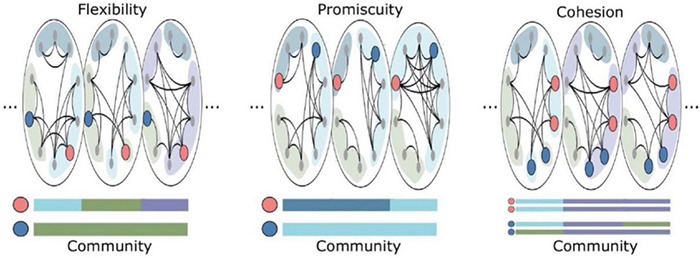
Reprinted with permissions from Sizemore and Bassett (2018). This schematic depicts examples of nodes with low (dark blue) or high (pink) flexibility, promiscuitiy, and cohesion. In the **(Left)** panel, the pink node is more flexible than the blue node (i.e., changes communities more often). In the **(Middle)** panel, the pink node has higher promiscuity than the blue node (i.e., visits a greater proportion of available communities across time). In the **(Right)** panel, the pink nodes have higher cohesion strength than the blue nodes (i.e., the pink nodes move between communities in tandem).

With respect to potential associations with consciousness, one fascinating study applied flexibility analyses to investigate dynamic alterations in the modular structure of nervous systems with varying depths of anesthesia (Standage et al., 2020). In this investigation, the anesthetic isoflurane was used to modulate consciousness level in rhesus macaques measured with high-field strength fMRI. In addition to examining disjoint and cohesive flexibility, an additional measure of promiscuity was utilized (Figure 5), calculated as the number of communities that a node participates in over time, divided by the total number of potentially available community allegiances (Sizemore and Bassett, 2018). In contrast to flexibility, promiscuity assesses the degree to which nodes take part in the full range of available communities, and so would speak to the “diverse club” findings described above for Yin et al. (2020).

**FIGURE 6:**
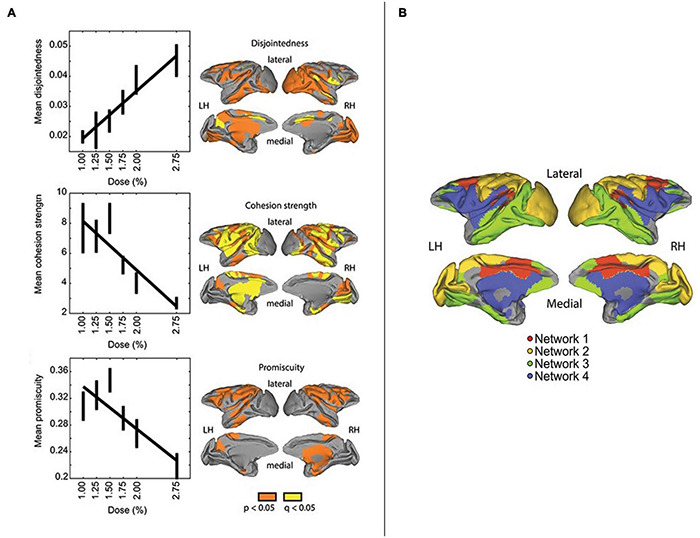
Reprinted with permissions from Standage et al. (2020). On the **(Left)**: relationship between isoflurane dose and mean disjointedness (top panel), mean cohesion strength (middle panel), and mean promiscuity (bottom panel) across the whole brain. The brain images directly next to these graphs show regions where the relationship between the dose and measure was significant (in orange), and significant with an additional false discovery rate correction (in yellow). On the **(Right)**: network architecture identified in the study, depicting four networks: 1/red = cingulate-temporal-parietal; 2/yellow = visual-somatomotor; 3/green = temporal-parietal-prefrontal; 4/blue = lateral parietal-frontal cingulate temporal.

Fascinatingly, Standage et al. (2020) observed that deeper sedation correlated with higher mean disjointedness, lower mean promiscuity, and lower mean cohesion strength (Figure 6). These findings potentially suggest that relatively high degrees of promiscuity/diversity might require cohesive dynamics to be realized. These patterns were further associated with greater network fragmentation, as evidenced by a larger number of communities with higher modularity values (interpreted as indicating functional isolation). Four functional networks were identified, corresponding to the cingulate-temporal-parietal, visual-somatomotor, temporal-parietal-prefrontal, and lateral parietal-frontal-cingulate-temporal areas. With greater degrees of sedation, these networks tended to become less distinct from each other, with visual-somatomotor areas constituting a notable exception in terms of maintained separability. Speculatively, this would make some sense given the close engagement of these areas with sense data, and potential (experience-dependent) development of more modular structure with greater locality in information processing.

Integrated Information Theory (IIT) may provide another means of assessing connections between network flexibility, criticality, and consciousness (Tononi et al., 2016; Safron, 2020, 2021). IIT was initially developed as a theory that started from the hypothesis that consciousness involves synergy, or wholes with informational properties that are greater than the sum of their parts (Tononi and Edelman, 1998). The theory was subsequently developed as a general model of emergent causation that analyzes (potentially conscious) systems in terms of their “irreducible self-cause-effect-power,” or capacity of present configurations to place informational constraints on their past and future states (Balduzzi and Tononi, 2009). The intuition underlying this modeling approach is that conscious systems are composed of “differences that make a difference” to themselves, or have intrinsic functional significance. Notably, integrated information appears to be maximized by systems that balance integration and differentiation, which is widely considered to be a prerequisite for adaptive complexity, and which has also been associated with connectomic properties stipulated to be necessary for realizing consciousness (Dehaene and Changeux, 2011; Shanahan, 2012; van den Heuvel et al., 2012; Sporns, 2013; Shine, 2019, 2021; Mashour et al., 2020; Safron, 2020, 2021).

While their potential sufficiency for establishing subjective experience is highly debatable, these analyses may nonetheless point to necessary conditions for realizing sufficiently complex (and thereby powerful) processing for realizing consciousness, potentially *via* cohesive flexibility. While IIT’s formal analyses are not computationally tractable for most biological networks (Mayner et al., 2018), a variety of approximations have been developed (Massimini et al., 2012; Tegmark, 2016; Mediano et al., 2019). Based on such estimates, integrated information appears to be maximized by systems that exhibit self-organized criticality (Arsiwalla and Verschure, 2016; Aguilera and Di Paolo, 2021). Further, if consciousness involves the flexible establishment of large-scale integrative modules (or “workspaces”), and if such complexes can only form for networks capable of establishing patterns of effective connectivity with balanced integration/differentiation—and order/stochasticity—then we ought to expect valid proxies of integrated information to correlate with such dynamic modularity. Speculatively, valid measures of modularity may provide computationally tractable estimates of integrated information if it is the case that such modules are only likely to self-assemble under conditions that allow for flexibly balanced dynamics. Finally, a recently proposed theory of consciousness has suggested connections between IIT’s “self-cause-effect-power” and a capacities of systems to generate themselves according to the general systems theory of the Free Energy Principle (Safron, 2020, 2021), which we will now discuss.

## Free Energy Minimization as Fundamental Principle of Brain Organization?

We will now review some fundamental principles of neuronal organization before going on to describe additional ways of characterizing complex networks of effective connectivity. What constitutes an appropriate explanation of neuronal assembly formation? It has been suggested that neuronal organization can be understood as the self-organization of boundaries in dynamical systems that minimize free energy (Friston, 2010, 2013).^3^ In this section we offer our view as to how the Free Energy Principle (FEP) can be employed within and to extend the dynamical approach we have been explicating so far, The FEP formalism explains the autonomous emergence of order in the brain as a dynamic self-assembling process (Parr et al., 2020, 2021; Friston et al., 2021).

We find systems that self-organize at every non-equilibrium scale (Friston, 2019). In order to persist, every thermodynamically-open system must self-organize as it exchanges matter and energy with the environment with which it is coupled (Palacios et al., 2017; Lamprecht and Zotin, 2019). That is, persisting systems must self-organize to (temporarily) defy the second law of thermodynamics and keep their (non-equilibrium) steady states from tending toward disorder and dissipation (Friston, 2013; Colombo and Palacios, 2021).

To avoid this maximally likely outcome of monotonically increasing disorder, systems must exhibit intelligent adaptivity in exchanging matter and energy with their environments. While some systems are simply subject to being entrained by environmental stochasticity and some dynamical rule, such as pendulums (Oliveira and Melo, 2015; Kirchhoff et al., 2018; Lahav et al., 2018), other systems can interact with their environment in order to achieve more desirable states for survival. Living systems such as cells, organs, and organisms, keep surprisal (i.e., cybernetic entropy) at bay by engaging in behavior that translates into stable minimal points (i.e., uncertainty minima) for a set of potential states within bounds that can mean life and death, respectively.

Evidence from dynamic causal modeling (Friston et al., 2019; Jafarian et al., 2021) provides compelling reasons to think that synaptic organization conforms with the conditional independence established by a Markov blanket on a dynamical setting (Friston et al., 2021; Hipólito et al., 2021b; Parr et al., 2021). A Markov blanket is a statistical tool that can be applied to any system that self-organizes. From particles and moving pendulums, to cells, neurons, brains, and organisms. By this formalism it is possible to partition a self-organizing system at every scale in terms of its conditionally independent states, that is, how the environment influences a system and vice versa. While the system of interest, e.g., a neuron, corresponds to the internal states, the environment (e.g., a cortical column of which the neuron is a part) the corresponds to the external states.

**FIGURE 7:**
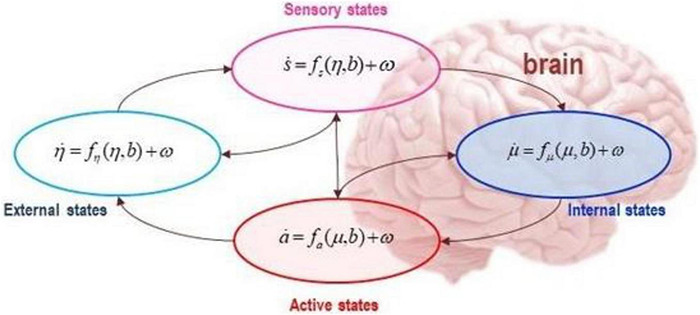
The figure depicts a Markov blanket partitioning of conditionally independent states, internal (blue) and external (cyan); and blanket states, active (red) and sensory (magenta), directly influencing one another. The arrows show that while internal states depend on blanket states (sensory and active states), external states depend on external states and blanket states. This means that internal and external states indirectly influence one another by virtue of the influences between blanket states: i.e., sensory and active states. The Markov blanket formalism is scale free, which means that it can be applied to any scale of the physical world to explain the exchanges and influences amongst open systems. Applied to the brain, the formalism can be applied such that internal states correspond to a single neuron, a cortical microcircuit, a region, or a network (see Hipólito et al., 2021b for a detailed application).

The Markov blanket formalism aims to determine how internal and external states influence each other. Mathematically we know that internal and external states do not directly influence one another (this is known as conditional independence), they instead indirectly influence one another by virtue of a further set of states: sensory and active states (known as blanket states) as shown in Figure 7. Technically, it is as if a neuron in cortical column (internal states) engages in predicting the state of the cortical column (external states) by issuing a prediction (active state), where the state of the cortical column (sensory state) directly influences the issuing prediction and *vice versa*.

The scale-free property of the Markov blanket formalism can be applied to every level of self-organization, from single neurons, to cortical columns, to brain regions, to mesoscale networks, to the entire nervous system, and beyond. In this way, it is possible to explain persisting systems as coupled with their multiscale environments at every level of organization. A system (i.e., internal states) can be seen as engaging in the required behavior of model-uncertainty-minimization by which intelligent systems adapt and maintain themselves. In other words, internal states appear to engage in behavior that reduces internal uncertainty or entropy and so avoids system dissipation. The ways in which the systems reduce uncertainty or entropy can be explained as if the system was minimizing a singular objective functional of informational free energy (i.e., accuracy minus complexity, or evidence with respect to the active inferential models whereby systems promote their existence).


(2) $$\underset{\mathrm{free}-\mathrm{energy}}{\underbrace{F\left(s,\mu \right)}}=\underset{\text{complexity}}{\underbrace{D_{\text{KL}}\left[q\left(\psi |\mu \right)|p\left(\psi |m\right)\right]}}-\underset{\text{accuracy}}{\underbrace{E_{q}\left[\log p\left(s|\psi ,m\right)\right]}}$$

Equation 2 represents free energy minimized with respect to internal and sensory states, corresponding to the difference between complexity and accuracy, or between the Kullback–Leibler (KL) divergence between the variational [*q*(ψ | μ) || *p* (ψ | *m*)], and the posterior density over hidden states *E*_*q*_[*log*⁡*p*(*s* | ψ, *m*)]. This framing of complex adaptive systems allows us to treat neuronal organization as an optimization problem. That is, how does the minimization of free energy ensure that brains optimize neuronal assemblies in ways that reduce entropy/uncertainty?

At every self-organizing scale of the brain, activity can be explained in terms of this sort of optimization. Brains, as dynamical systems, can be cast as minimizing variational free energy with respect to the sufficient statistics $\tilde{q}$ of an approximate posterior distribution $q(\theta |\tilde{q}$) (Roweis and Ghahramani, 1999; Friston, 2008). Under the Laplace approximation, these sufficient statistics correspond to the mean and covariance of probabilistic densities. The adaptive shaping of neuronal activity thus becomes an optimization problem (as opposed to an inference problem) (Dayan et al., 1995) with respect to implicit generative models governing system evolution:


(3) $$\tilde{q}=\arg max_{q}F\left(\tilde{p}|\tilde{q}\right)$$

This optimization with respect to (implicit) probabilistic densities corresponds to the minimization of free energy given the prior $\tilde{p}$, such that,


(4) $$F\left(\tilde{p,}\tilde{q}\right)=E_{q}\left[lnp\left(y|\theta \right)\right]-D_{KL}\left[q\left(\theta |\tilde{q}\right)||p\left(\theta |\tilde{p}\right)\right]$$

Free energy minimization [$F\left(\tilde{p,}\tilde{q}\right)$] is expressed in terms of accuracy (first term) minus complexity (second term), which is also the Kullback-Leibler divergence between the approximate posterior ($\tilde{q}$) and prior ($\tilde{p}$) distributions. After the negative free energy has been maximized, the following approximate inequalities can be used to estimate the posterior density over unknown model parameters, as well as the log evidence, or (marginal) likelihood of the model:


(5) $$q\left(\theta |{\tilde{q}}^{*}\right)\approx p\left(\theta |y,\tilde{p}\right)$$

$$F\left(\tilde{p},{\tilde{q}}^{*}\right)\approx lnp\left(y|\tilde{p}\right)$$

This means that the approximate posterior over parameters is a functional of the approximate posterior for actions inferred for present and future states. This informational free energy corresponds to the inverse probability of predictive responses—as a joint mapping between hidden states and observations, thus specifying a generative model—given an approximate posterior. The free energy gradient (and entailed dynamical fluctuations) are minimal when the average differences between posterior and prior expectations is zero, thus specifying the extent of systems as non-equilibrium steady state distributions.

## On Particles and Parcels: Dynamic Causal Modeling, Markov Blankets, and Dynamic Modularity

Regardless of whether or not one is compelled by the free energy perspective, this research program has generated a number of powerful analytic techniques. Before returning to more commonly known approaches for characterizing dynamic brain networks, we will briefly explore additional methods for assessing emergent modularity, which may lead to advances in both theoretical and applied domains. These analyses could potentially be applied alongside other measures to provide more detailed accounts of the latent processes that generate neuroimaging data. This cross-referencing of methods could further provide enhanced interpretability of datasets, and potentially help to inspire the development of novel analytic tools.

Renormalisation Group (RG) theory provides a principled account of dimensionality reduction in complex systems. Applied to the brain as a set of dynamically evolving Markov blankets, it offers formalisms for moving up and down analytical—and perhaps ontological (van Es and Hipolito, 2020)—levels depending on our area of interest for scientific description. In doing so, this approach affords understanding neuronal dynamics across a range of (nested) spatial and temporal scales (Friston et al., 2021).

States partitioned by the Markov blanket are multidimensional vector states, or eigenvectors. From these eigenvectors, by means of the RG theory, it is then possible to construct new states at a superordinate scale. We proceed by considering the principal eigenvectors of the blanket states and take those as eigenstates for the scale above. The recursive application of a grouping or partition operator (G), followed by dimensionality reduction (R), allows us to define a renormalisation group. Put simply, this method allows us to scale up across levels of emergent/synergistic organization, with subsequent levels characterized by more encompassing scopes of integration, evolving with increasingly slow/stable temporal dynamics. The dimension operator (R) allows us to eliminate (1) the internal states (as these, by definition, do not contribute to coupling) and (2) fast eigenstates (unstable or fast modes of a dynamical system that more rapidly dissipate). These two simple operations allow us to retain only slow and stable eigenvalues, which we can see as an adiabatic approximation to separate out fast and slow dynamics. This separation rests on the eigenvectors of the Jacobian for each Markov blanket, with eigenvectors separated into small (slow) and large negative (fast) eigenvalues:


(6) $$\begin{array}{c} \left[\begin{array}{ccc} \lambda_{11}^{\left(i\right)} & & \lambda_{1J}^{\left(i\right)}\\ \lambda_{J1}^{\left(i\right)} & & \lambda_{JJ}^{\left(i\right)} \end{array}\right]=\left[\begin{array}{cc} \begin{array}{c} \xi_{1}^{\left(i\right)}\\ \xi_{j}^{\left(i\right)} \end{array} & \begin{array}{c} \zeta_{1}^{\left(i\right)}\\ \zeta_{j}^{\left(i\right)} \end{array} \end{array}\right]\left[\begin{array}{ccc} J_{11} & & J_{11}\\ J_{J1} & & J_{JJ} \end{array}\right]\left[\begin{array}{cc} \begin{array}{c} \xi_{1}^{\left(i\right)}\\ \xi_{J}^{\left(i\right)} \end{array} & \begin{array}{c} \zeta_{1}^{\left(i\right)}\\ \zeta_{J}^{\left(i\right)} \end{array} \end{array}\right]\\ \lambda_{jj}^{\left(i\right)}={\left[\xi_{j}^{\left(i\right)},\zeta_{j}^{\left(i\right)}\right]}^{-}J_{jj}\left[\xi_{j}^{\left(i\right)},\zeta_{j}^{\left(i\right)}\right]=\left[\begin{array}{cc} \lambda {\_}_{jj}^{\xi \xi } & 0\\ 0 & \lambda_{jj}^{\zeta \zeta } \end{array}\right]\\ \lambda_{jk}^{\left(i\right)}={\left[\xi_{j}^{\left(i\right)},\zeta_{j}^{\left(i\right)}\right]}^{-}J_{jk}\left[\xi_{k}^{\left(i\right)},\zeta_{k}^{\left(i\right)}\right]=\left[\begin{array}{cc} \lambda {\_}_{jk}^{\xi \xi } & \lambda {\_}_{jk}^{\xi \xi }\\ \lambda {\_}_{jk}^{\xi \xi } & \lambda_{jk}^{\zeta \zeta } \end{array}\right]\\ {\left[\xi_{j}^{\left(i\right)},\zeta_{j}^{\left(i\right)}\right]}^{-}\left[\xi_{j}^{\left(i\right)},\zeta_{j}^{\left(i\right)}\right]=1,0\geq Re\lambda_{jj}^{\xi \xi }>\epsilon \geq Re\lambda_{jj}^{\zeta \zeta } \end{array}$$

As an adiabatic reduction (Haken, 1983), with its related manifold theorem (Carr, 1981), we can see that dynamics get progressively slower at successive scales: where the intrinsic coupling among eigenstates is constituted by a diagonal matrix of (negative) eigenvalues, so determining the relative decay of multiscale attractors,


(7) $$E\left[Re\left(\lambda_{nn}^{\left(i\right)}\right)\right]\leq E\left[Re\left(\lambda_{nn}^{\left(i+1\right)}\right)\right]\ldots \leq 0$$

**FIGURE 8:**
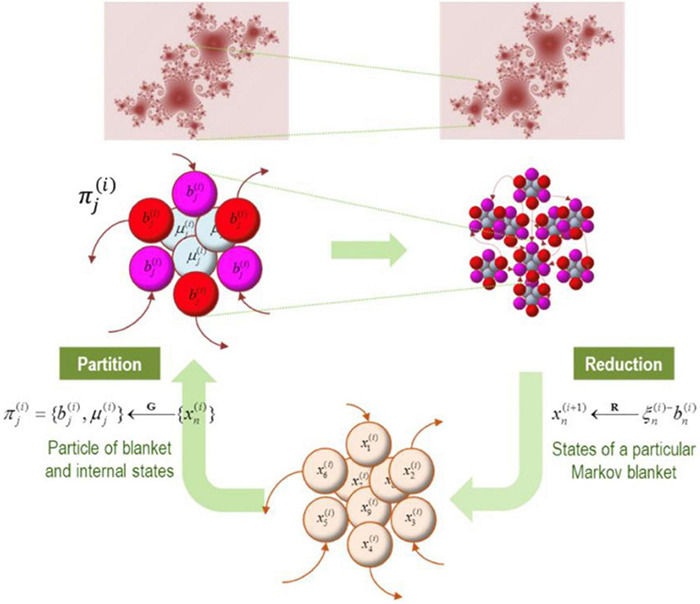
In this figure we have an illustration of progressively larger scales (and slower dynamics) arising from subordinate levels. In the upper panels, the conditional dependencies among these vector states (i.e., eigenstates) define a particular partition into particles. This partition then equips each particle with a bipartition into blanket and internal states, where blanket states comprise active (red) and sensory (magenta) states. The behavior of each particle can now be summarized in terms of (slow) eigenstates or mixtures of its blanket states to produce states at the next level or scale. Vector states (i.e., eigenstates) on the bottom can be partitioned into particles (upper panels). Each particle can then be partitioned into internal and blanket states, which involve active (red) and sensory states (magenta). The behavior of each particle can be summarized either as (slow) eigenstates or mixtures of its blanket states to produce states to the next level or scale (i.e., an ensemble of vector states). Note that the first uses the particular partition to group subsets of states (G); while the second uses eigenstates of the resulting blanket states to reduce dimensionality (R) (Figure reproduced from Friston, 2019).

In short, we can eliminate fast eigenstates and approximate dynamics with the remaining slow eigenstates that capture the dynamics ‘that matter’ for explaining large-scale system properties. Importantly, as shown in Figures 7, 8, these renormalized flows allow us to progress from more chaotic high amplitude dynamics to more deterministic (and potentially interpretable) dynamics of slow fluctuations that are likely to dominate overall system evolution (Friston et al., 2012, 2014). This simply means that we are adopting a common procedure known as ‘dimensionality reduction.’ The more complex (i.e., higher dimensional) the system, the harder it is to mathematically track its dynamics. Progressing from higher to more deterministic amplitude also means that things become more interpretable.

**FIGURE 9:**
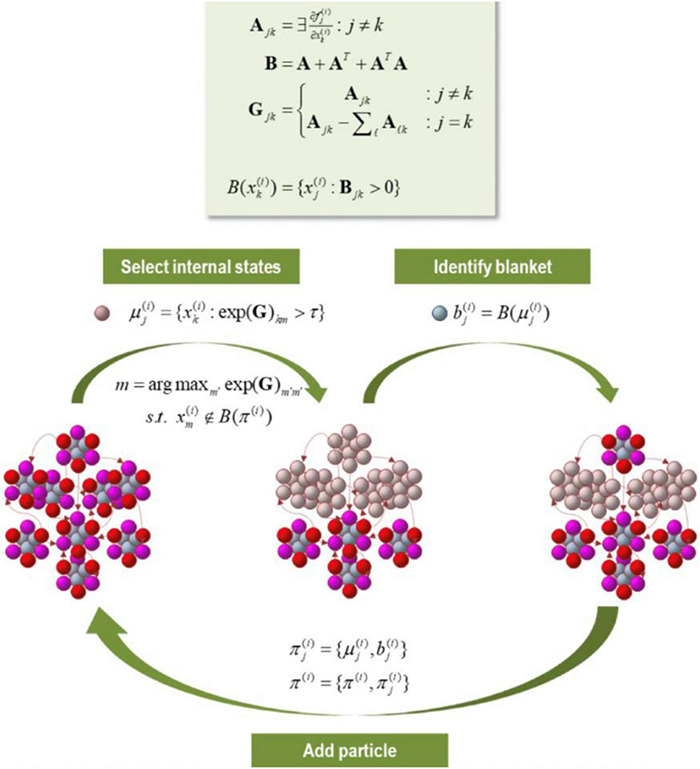
In this figure we have a partition of eigenstates (small colored balls) into particles, where each particle displays 6 blanketed states (active states in red; sensory states in magenta), and 3 internal states (cyan). Based upon the Jacobian and implicit flow of vector states, an adjacency matrix characterizes the coupling between vector states, which defines the blanket forming matrix (B), and to form a Laplacian (G) that is used to define coupled internal states. The internal and blanket states then form a new particle. The procedure is exhausted when unassigned vector states belong to the Markov blanket of the particles identified previously.

The Jacobian in Figure 9 summarizes effective connectivity at the smallest scale, so allowing us to investigate intrinsic dynamics at progressively larger scales. Lyapunov exponents are the same as the eigenvalues of the Jacobian describing intrinsic coupling. By associating the Jacobian of each particle with Lyapunov exponents, it is possible to score the average exponential rate of divergence or convergence of trajectories in state space (Lyapunov, 1992; Yuan et al., 2011; Pavlos et al., 2012). There is a progressive slowing of intrinsic dynamics as we move up the dynamics at larger (higher) scales toward critical regimes of instability and slowly fluctuating dynamical modes. As previously discussed with respect to self-organized criticality, the importance of such unstable/metastable regimes may be difficult to overstate, and analysis of Lyapunov exponents for signs of critical slowing provides a powerful means of assessing such adaptive organization. This also brings us to a notable point about the brain: particles that constitute the active (gray) matter, when considered in isolation, show autonomous dynamics that can be cast in terms of stochastic chaos or itinerancy. Autonomous neural dynamics emerge as characteristics associated with intrinsic connectivity (where Lyapunov exponents of the intrinsic coupling describe the rate of decay). The extrinsic dynamics concerns, however, the extent to which one eigenstate influences another. Of crucial interest here is rate constants, or the degree to which an eigenstate of one particle responds to the eigenstate of another. Notably, large extrinsic couplings can also be understood as cross-variance functions, or a kind of implicit form of ‘representation.’

In conclusion, the application of the RG formalism on states partitioned by Markov blankets allows us to see the emergence of order in neuronal activity as intrinsic connectivity (intrinsic dynamics) and dynamic coupling (extrinsic dynamics). Every state, at this multiscale system, organizes to keep surprisal at bay. In other words, the action of all complex adaptive systems can be explained as self-organizing to minimize free energy, and so persist through intelligent active inference. Evolutionary predispositions (Badcock et al., 2019), together with stochastic forcing from environmental pressures, canalize dynamics to enact certain trajectories relative to others, corresponding to the emergence of neuronal assemblies capable of functioning as (potentially flexible) attracting manifolds for adaptive functioning (Friston et al., 2020).

## Future Directions for Understanding Flexibility in Brains and Minds

One limitation of methods for assessing network flexibility (and for connectomics more generally) is an assumption of dichotomous in/out properties with respect to community membership. Theoretically, community allegiance could be made a matter of degree—some of which may also represent differences in kind (Anderson, 1972)—with flexibility weighted by degrees of modularity, which the Generalized Louvain algorithm quantifies as Q (for “quality,” or extent of preferential inner-connectivity for a community) (Jutla et al., 2011). This algorithm is computationally efficient and empirically valid, and also has face validity in terms of modularity being important for allowing for separable optimizations of sub-systems with potentially fewer functional tradeoffs. However, these methods treat modules in overly simplistic ways, and neglect to consider the extent to which nodes can participate in multiple communities to varying degrees, potentially involving functional multiplexing with multiscale organization.

There are other more powerful module detection methods like stochastic block models, which use generative modeling to infer community structure (Lee and Wilkinson, 2019). But these are computationally expensive, and so are less frequently utilized. Infomap is another community-detection method, which estimates communities using random walks and relative dwell times as indicating the degree of modularity for an area (Rosvall and Bergstrom, 2008). This method has correspondences with early versions of Google’s PageRank algorithm, and also has connections to percolation methods (Mišić et al., 2015), both of which could be used to reflect overlapping community structures.

**FIGURE 10:**
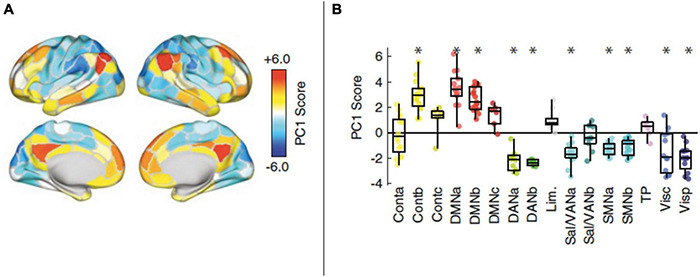
**(A,B)** Networks identified as contributing to the high-amplitude co-fluctuations. Positive contributions were indicated by high PC1 scores, with significant contributions made by default mode areas (within DMNa and DMNb) or control areas (within Contb). On the right, asterisks correspond to PC1 scores obtained in a principal component analysis (performed on a correlation matrix not shown in this modified figure). Further details describing the principal component analysis used to obtain these results can be found in Esfahlani et al., 2020. Reprinted with permission from Esfahlani et al. (2020).

Edge-centric time-series are also promising in supporting multiscale/multiplexed accounts. One notable study involving these methods was able to analyze dynamics at the temporal scale of a single fMRI measurement (2000 ms), and found that transient high-amplitude co-fluctuations in cortical activity contributed to overall patterns of connectivity (Esfahlani et al., 2020). These events occurred at variable intervals (Figure 10), sometimes closely spaced, and sometimes separated by tens of seconds or even minutes. Areas contributing to these high-amplitude highly-impactful events included default mode and control network areas. Although anatomical regions were not discussed in this study, these networks include the temporoparietal junction, posterior cingulate, precuneus, as well as dorsomedial, ventromedial, premotor, dorsolateral, and temporal cortices. In other words, with the exception of the precuneus, the most impactful areas of the brain on overall dynamics may be centered on the previously described “flexible club” of the brain. More recent work has identified additional clusters that contribute to high-amplitude cofluctuations; however, the DMN still appears to be a consistent contributor to these events (Betzel et al., 2021). The authors describe these events as indicating “a high-modularity brain state and… a specific mode of brain activity, in which default mode and control networks fluctuate in opposition to sensorimotor and attention systems.” Notably, this functional alternation is a hallmark of conscious processing—and potentially workspace dynamics (Safron, 2020, 2021)—and is disrupted in neuropsychiatric conditions (Huang et al., 2020). Fascinatingly, these occurrences also corresponded to periods where participants were most readily uniquely identified using connectomic “fingerprinting” methods. Theoretically, these events could correspond to periods associated with particularly high meaningfulness (Li et al., 2021), with concomitant release of neuromodulators influencing the relative dominance of different functional networks (Shine et al., 2018; Conio et al., 2020).

Perhaps even more intriguingly, these brain areas associated with particularly high flexibility also tend to emerge later in phylogeny, mature later in ontogeny, and exhibit reduced degrees of structure-function tethering, implying greater degrees of freedom (Oligschläger et al., 2019; Vázquez-Rodríguez et al., 2019; Baum et al., 2020). Theoretically, expansion of these areas may have substantially contributed to the evolution of uniquely human cognition, with its capacity for flexible and creative high-level reasoning (Penn et al., 2008; Gentner, 2010; Buckner and Krienen, 2013; Hofstadter and Sander, 2013). It will be exciting to see whether such hypotheses are supported (or refuted) by future work with comparative neuroanatomy between human and non-human species (van den Heuvel et al., 2019; Changeux et al., 2020; Dumas et al., 2021).

**FIGURE 11:**
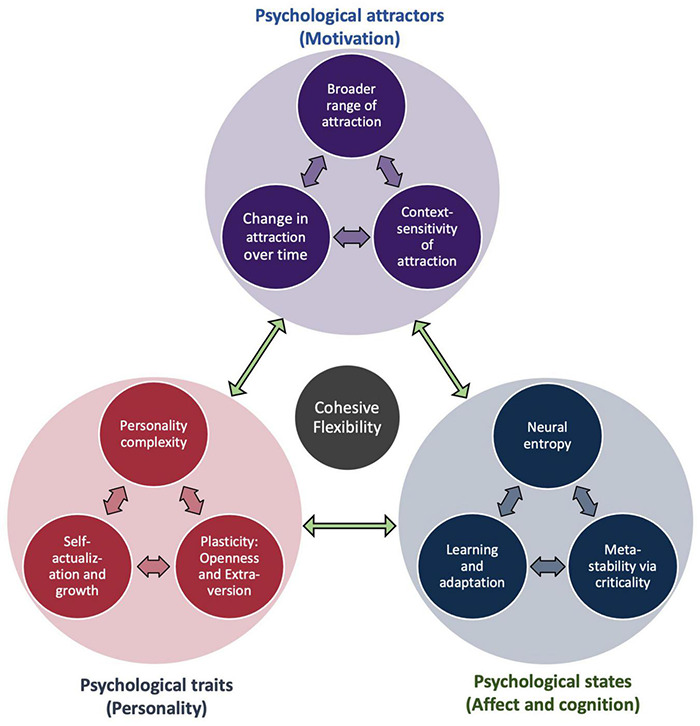
Cohesive flexibility and multi-scale psychology. This diagram illustrates how cohesive flexibility may provide a functional bridge between moment-to-moment changes in psychological states, more enduring psychological traits, and the attracting states by which individuals evolve through time as cybernetic (free energy minimizing) systems. At each level of organization, flexible (potentially self-organized critical) dynamic processes allow for intelligent responding, learning, and evolution toward increasing degrees of adaptive complexity.

In order to better characterize these kinds of dynamics and their potential functional significance for adaptive functioning, it would be highly desirable to undertake a systematic investigation into the following question: to what extent do different kinds of psychological flexibility correlate with different kinds of neural flexibility? For example, might the extent to which brain networks are capable of reconfiguring themselves due to a change in state [e.g., as induced by pharmacological agents (Doss et al., 2021; Girn et al., 2021)] also impact capacities for change with respect to more enduring traits (e.g., personality structures) (Figure 11)? In which ways might younger individuals exhibit more flexible brain dynamics, and might this relate to cognitive flexibility and more exploratory approaches to searching through hypothesis spaces (Gopnik et al., 2017)? If cohesive flexibility is indeed a hallmark of self-organized criticality as we have previously suggested, then we ought to expect it to manifest across multiple scales, ranging from moment-to-moment dynamic alterations of internal states, to the attractors describing trajectories of entire systems through phase space, and even the overall character of systems as shaped by histories of experience (Safron and DeYoung, 2021). In this way, the conditions under which individuals exhibit different forms of flexible dynamics may have far reaching implications with respect to the project of furthering computational psychiatry and precision medicine (Friston et al., 2017b). Finally, given the multiple scales over which such phenomena may evolve, analyzing the correlates of psychological flexibility is a near perfect use case for implementing the previously described multiscale methods for characterizing dynamical systems in terms of their probabilistic boundaries (Friston et al., 2021).

## Conclusion

In this theoretical review, we have considered brains from a dynamical perspective, discussed ways that such systems could be analyzed with a variety of neuroimaging techniques, and considered their potential grounding in relevant mechanistic processes and formal models. We hope this discussion will help generate enthusiasm for adopting a more dynamic perspective in attempting to understand the emergence of mental phenomena from biophysical processes. It may be difficult to overstate the importance of network flexibility for not just basic, but also applied sciences, since psychological flexibility constitutes a general factor for resilience across both clinical and non-clinical contexts (Hinton and Kirmayer, 2017; Hayes, 2019; Davis et al., 2020; Uddin, 2021). Perhaps more fundamentally, there may be few things more impactful than obtaining a better understanding of the factors contributing to the abilities of systems to adapt in a complex, uncertain, and constantly changing world.

## Author Contributions

All authors listed have made a substantial, direct, and intellectual contribution to the work, and approved it for publication.

## Conflict of Interest

The authors declare that the research was conducted in the absence of any commercial or financial relationships that could be construed as a potential conflict of interest.

## Publisher’s Note

All claims expressed in this article are solely those of the authors and do not necessarily represent those of their affiliated organizations, or those of the publisher, the editors and the reviewers. Any product that may be evaluated in this article, or claim that may be made by its manufacturer, is not guaranteed or endorsed by the publisher.

## Footnotes

1 For terminology conventionally employed in Dynamical Systems Theory, please refer to the Glossary.

2 A saddle point or minimax point is a point on the surface of the graph of a function where the slopes (derivatives) in orthogonal directions are all zero (a critical point), but which is not a local extremum of the function.

3 Please refer to the Glossary for terminology conventionality used in the Free Energy Principle.

## Glossary

**Glossary of terminology conventionally employed in Dynamical Systems Theory.**

**Free energy principle:** A principle that states how natural systems remain under non-equilibrium steady conditions by restricting themselves to a limited number of states. Living systems maintain homeostasis by minimizing variational free energy. The evolution of systems is explained in terms of free energy minimization by the internal states of the system, which is formally related to variational Bayesian methods, also known as active inference.

**Active inference:** A “first principles” approach to understanding behavior and the brain, framed in terms of a single imperative to minimize free energy. A system’s behavior is explained as if it engages in active inference such that internal states minimize free energy given a generative model.

**Generative model:** A generative model here refers to a probabilistic model of external states that influences Markov blanket boundaries, which are implicit in the dynamics of internal states.

**Markov blankets:** Markov blankets are the cornerstone of variational approaches of self-organization under the free energy principle. In the context of active inference, a Markov blanket is a statistical tool that allows an open/coupled system to be partitioned into internal and external states by virtue of another set of states called blanket states comprising sensory and active states. Mathematically, internal and external states indirectly influence one another by virtue of blanket states.

**Free energy minimization:** Casting the minimisation of thermodynamic free energy in terms of variational free energy allows one to interpret (the dynamics of) a system as minimisation of uncertainty, entropy, or surprisal.

**(Statistical) surprisal:** The term surprisal should not be confused with psychological surprise. It is a statistical term that refers to the “surprise” of seeing the outcome (a highly improbable outcome is very surprising). This means minimizing surprise maximizes the evidence for the agent (model). Put simply, the agent becomes a model of the environment in which it is immersed.

**Posterior/approximate posterior:** The idea of variational bayes is to construct an analytical approximation to the posterior probability of the set of unobservable variables (parameters and latent variables), given data. This is a useful method in computational modeling for dimensionality reduction (reducing non-linearity to linearity) of brain data. A model of brain data is constructed in order to explain how data was generated (unobservable or hidden variables).

**Laplace approximation:** The Laplace approximation framework aims to find a Gaussian approximation to a continuous distribution. It is the simplest deterministic method for approximate inference. It consists in approximating a posterior distribution with a Gaussian centered at the maximum *a posteriori* (MAP) estimate. This is the application of Laplace’s method with *f*(θ) = *p*(λ\θ)*p*(θ). This can be justified by the fact that under certain regularity conditions, the posterior distribution approaches a Gaussian as the number of samples grow.

**Probability density function (pdf):** Function whose value at any given sample (or point) in the sample space (the set of possible values taken by the random variable) can be interpreted as providing a relative likelihood that the value of the random variable would equal that sample.

**Entropy:** A thermodynamic quantity representing the unavailability of a system’s thermal energy for conversion into mechanical work, often interpreted as the degree of disorder or randomness in the system.

**Dynamical system:** A system in which a function describes the time dependence of a point in a geometrical space. Examples include the mathematical models that describe the swinging of a clock pendulum, the flow of water in a pipe, and the number of fish changing with seasons in a lake, or a model of neuronal activity as a complex system.

**Complex system:** A system with large degrees of freedom that has distinct properties that arise or emerges from relationships, such as non-linearity, emergence, spontaneous order, adaptation, and feedback loops, among others.

**Degrees of freedom:** In statistics, the number of degrees of freedom is the number of values in the final calculation of a statistic that are free to vary.

**Differential equation:** A differential equation is a mathematical relationship between a function and its derivatives. It can describe how populations change, how heat moves, how springs vibrate, how radioactive material decays and much more.

**Fixed point attractors:** An attractor is a set of states toward which a system tends to evolve, for a wide variety of starting conditions of the system. System values that get close enough to the attractor values remain close even if slightly disturbed.

**Limit cycles:** A closed trajectory in phase space having the property that at least one other trajectory spirals into it either as time approaches infinity or as time approaches negative infinity.

**Phase transition:** Physical processes of transition between a state of a medium, identified by some parameters, and another one, with different parameter values.

## References

[B1] Afraimovich V. Tristan I. Huerta R. Rabinovich M. I. (2008). Winnerless competition principle and prediction of the transient dynamics in a Lotka–Volterra model. *Chaos* 18:043103. 10.1063/1.2991108 19123613

[B2] Aguilera M. Di Paolo E. A. (2021). Critical integration in neural and cognitive systems: beyond power-law scaling as the hallmark of soft assembly. *Neurosci. Biobehav. Rev.* 123 230–237. 10.1016/j.neubiorev.2021.01.009 33485887

[B3] Anderson P. W. (1972). More is different. *Science* 177 393–396. 10.1126/science.177.4047.393 17796623

[B4] Arbib M. A. Arbib F. J. P. Arbib P. H. (2003). *The Handbook of Brain Theory and Neural Networks.* Cambridge, MA: MIT Press.

[B5] Arsiwalla X. D. Verschure P. F. M. J. (2016). “High integrated information in complex networks near criticality,” in *Artificial Neural Networks and Machine Learning – ICANN 2016*, eds Villa A. E. P. Masulli P. Pons Rivero A. J. (Cham: Springer International Publishing), 184–191.

[B6] Atasoy S. Deco G. Kringelbach M. L. (2019). “Playing at the edge of criticality: expanded whole-brain repertoire of connectome-harmonics,” in *The Functional Role of Critical Dynamics in Neural Systems*, eds Tomen N. Herrmann J. M. Ernst U. (Cham: Springer International Publishing), 27–45. 10.1007/978-3-030-20965-0_2

[B7] Atasoy S. Roseman L. Kaelen M. Kringelbach M. L. Deco G. Carhart-Harris R. L. (2017). Connectome-harmonic decomposition of human brain activity reveals dynamical repertoire re-organization under LSD. *Sci. Rep.* 7:17661. 10.1038/s41598-017-17546-0 29247209 PMC5732294

[B8] Badcock P. B. Friston K. J. Ramstead M. J. D. (2019). The hierarchically mechanistic mind: a free-energy formulation of the human psyche. *Phys. Life Rev.* 31 104–121. 10.1016/j.plrev.2018.10.002 30704846 PMC6941235

[B9] Bak P. Tang C. Wiesenfeld K. (1987). Self-organized criticality: an explanation of the 1/f noise. *Phys. Rev. Lett.* 59 381–384. 10.1103/PhysRevLett.59.381 10035754

[B10] Balduzzi D. Tononi G. (2009). Qualia: the geometry of integrated information. *PLoS Comput. Biol.* 5:e1000462. 10.1371/journal.pcbi.1000462 19680424 PMC2713405

[B11] Bassett D. S. Wymbs N. F. Porter M. A. Mucha P. J. Carlson J. M. Grafton S. T. (2011). Dynamic reconfiguration of human brain networks during learning. *Proc. Natl. Acad. Sci. U.S.A.* 108 7641–7646. 10.1073/pnas.1018985108 21502525 PMC3088578

[B12] Bastos A. M. Lundqvist M. Waite A. S. Kopell N. Miller E. K. (2020). Layer and rhythm specificity for predictive routing. *Proc. Natl. Acad. Sci. U.S.A.* 117 31459–31469. 10.1073/pnas.2014868117 33229572 PMC7733827

[B13] Baum G. L. Cui Z. Roalf D. R. Ciric R. Betzel R. F. Larsen B. (2020). Development of structure–function coupling in human brain networks during youth. *Proc. Natl. Acad. Sci.* 117 771–778. 10.1073/pnas.1912034117 31874926 PMC6955327

[B14] Beaty R. E. Benedek M. Barry Kaufman S. Silvia P. J. (2015). Default and executive network coupling supports creative idea production. *Sci. Rep.* 5:10964. 10.1038/srep10964 26084037 PMC4472024

[B15] Beaty R. E. Kaufman S. B. Benedek M. Jung R. E. Kenett Y. N. Jauk E. (2016). Personality and complex brain networks: the role of openness to experience in default network efficiency. *Hum. Brain Mapp.* 37 773–779. 10.1002/hbm.23065 26610181 PMC4738373

[B16] Bechtel W. Graham G. (1998). *A Companion to Cognitive Science.* Hoboken, NJ: Wiley.

[B159] Betzel R. F. Cutts S. A. Greenwell S. Faskowitz J. Sporns O. (2021). Individualized event structure drives individual differences in whole-brain functional connectivity. *bioRxiv* 10.1101/2021.03.12.435168 35192942

[B17] Betzel R. F. Satterthwaite T. D. Gold J. I. Bassett D. S. (2017). Positive affect, surprise, and fatigue are correlates of network flexibility. *Sci. Rep.* 7:520. 10.1038/s41598-017-00425-z 28364117 PMC5428446

[B18] Braun U. Schäfer A. Bassett D. S. Rausch F. Schweiger J. I. Bilek E. (2016). Dynamic brain network reconfiguration as a potential schizophrenia genetic risk mechanism modulated by NMDA receptor function. *Proc. Natl. Acad. Sci. U.S.A.* 113 12568–12573. 10.1073/pnas.1608819113 27791105 PMC5098640

[B19] Braun U. Schäfer A. Walter H. Erk S. Romanczuk-Seiferth N. Haddad L. (2015). Dynamic reconfiguration of frontal brain networks during executive cognition in humans. *Proc. Natl. Acad. Sci. U.S.A.* 112 11678–11683. 10.1073/pnas.1422487112 26324898 PMC4577153

[B20] Buckner R. L. Krienen F. M. (2013). The evolution of distributed association networks in the human brain. *Trends Cogn. Sci.* 17 648–665. 10.1016/j.tics.2013.09.017 24210963

[B21] Campbell J. O. (2016). Universal Darwinism as a process of Bayesian inference. *Front. Syst. Neurosci.* 10:49. 10.3389/fnsys.2016.00049 27375438 PMC4894882

[B22] Carhart-Harris R. L. Leech R. Hellyer P. J. Shanahan M. Feilding A. Tagliazucchi E. (2014). The entropic brain: a theory of conscious states informed by neuroimaging research with psychedelic drugs. *Front. Hum. Neurosci.* 8:20. 10.3389/fnhum.2014.00020 24550805 PMC3909994

[B23] Carr J. (1981). *Applications of Centre Manifold Theory*, Vol. 35. New York, NY: Springer US. 10.1007/978-1-4612-5929-9

[B24] Chai L. R. Mattar M. G. Blank I. A. Fedorenko E. Bassett D. S. (2016). Functional network dynamics of the language system. *Cereb. Cortex* 26 4148–4159. 10.1093/cercor/bhw238 27550868 PMC5066829

[B25] Chang D. Song D. Zhang J. Shang Y. Ge Q. Wang Z. (2018). Caffeine caused a widespread increase of resting brain entropy. *Sci. Rep.* 8:2700. 10.1038/s41598-018-21008-6 29426918 PMC5807546

[B26] Changeux J.-P. Goulas A. Hilgetag C. C. (2020). Feature article: a connectomic hypothesis for the hominization of the brain. *Cereb. Cortex* 31 2425–2449. 10.1093/cercor/bhaa365 33367521 PMC8023825

[B27] Cieri F. Zhuang X. Caldwell J. Z. K. Cordes D. (2021). Brain entropy during aging through a free energy principle approach. *Front. Hum. Neurosci.* 15:647513. 10.3389/fnhum.2021.647513 33828471 PMC8019811

[B28] Colombo M. Palacios P. (2021). Non-equilibrium thermodynamics and the free energy principle in biology. *Biol. Philos.* 36:41. 10.1007/s10539-021-09818-x

[B29] Conio B. Martino M. Magioncalda P. Escelsior A. Inglese M. Amore M. (2020). Opposite effects of dopamine and serotonin on resting-state networks: review and implications for psychiatric disorders. *Mol. Psychiatry* 25 82–93. 10.1038/s41380-019-0406-4 30953003

[B30] Damicelli F. Hilgetag C. C. Hütt M.-T. Messé A. (2019). Topological reinforcement as a principle of modularity emergence in brain networks. *Netw. Neurosci.* 3 589–605. 10.1162/netn_a_00085 31157311 PMC6542620

[B31] Daunizeau J. David O. Stephan K. E. (2011). Dynamic causal modelling: a critical review of the biophysical and statistical foundations. *Neuroimage* 58 312–322. 10.1016/j.neuroimage.2009.11.062 19961941

[B32] Davey C. G. Harrison B. J. (2018). The brain’s center of gravity: how the default mode network helps us to understand the self. *World Psychiatry* 17 278–279. 10.1002/wps.20553 30192084 PMC6127769

[B33] Davis A. K. Barrett F. S. Griffiths R. R. (2020). Psychological flexibility mediates the relations between acute psychedelic effects and subjective decreases in depression and anxiety. *J. Contextual Behav. Sci.* 15 39–45. 10.1016/j.jcbs.2019.11.004 32864325 PMC7451132

[B34] Davis T. Poldrack R. A. (2013). Measuring neural representations with fMRI: practices and pitfalls. *Ann. N. Y. Acad. Sci.* 1296 108–134. 10.1111/nyas.12156 23738883

[B35] Dayan P. Hinton G. E. Neal R. M. Zemel R. S. (1995). The Helmholtz machine. *Neural Comput.* 7 889–904. 7584891 10.1162/neco.1995.7.5.889

[B36] Deco G. Jirsa V. K. (2012). Ongoing cortical activity at rest: criticality, multistability, and ghost attractors. *J. Neurosci.* 32 3366–3375. 10.1523/JNEUROSCI.2523-11.2012 22399758 PMC6621046

[B37] Dehaene S. Changeux J.-P. (2011). Experimental and theoretical approaches to conscious processing. *Neuron* 70 200–227. 10.1016/j.neuron.2011.03.018 21521609

[B38] Doss M. K. Považan M. Rosenberg M. D. Sepeda N. D. Davis A. K. Finan P. H. (2021). Psilocybin therapy increases cognitive and neural flexibility in patients with major depressive disorder. *Transl. Psychiatry* 11 1–10. 10.1038/s41398-021-01706-y 34750350 PMC8575795

[B39] Dumas G. Malesys S. Bourgeron T. (2021). Systematic detection of brain protein-coding genes under positive selection during primate evolution and their roles in cognition. *Genome Res.* 31 484–496. 10.1101/gr.262113.120 33441416 PMC7919455

[B40] Esfahlani F. Z. Jo Y. Faskowitz J. Byrge L. Kennedy D. P. Sporns O. (2020). High-amplitude co-fluctuations in cortical activity drive functional connectivity. *bioRxiv* [Preprint] 10.1101/800045 PMC7668041 33093200

[B41] Favela L. H. (2020). The dynamical renaissance in neuroscience. *Synthese* 199 2103–2127. 10.1007/s11229-020-02874-y

[B42] Freeman W. (1975). *Mass Action in the Nervous System.* New York, NY: Elsevier. 10.1016/C2009-0-03145-6

[B43] Freeman W. J. Kozma R. (2010). Freeman’s mass action. *Scholarpedia* 5:8040. 10.4249/scholarpedia.8040

[B44] Friston K. Breakspear M. Deco G. (2012). Perception and self-organized instability. *Front. Comput. Neurosci.* 6:44. 10.3389/fncom.2012.00044 22783185 PMC3390798

[B45] Friston K. J. (2008). Hierarchical models in the brain. *PLoS Comput. Biol.* 4:e1000211. 10.1371/journal.pcbi.1000211 18989391 PMC2570625

[B46] Friston K. J. (2010). The free-energy principle: a unified brain theory? *Nat. Rev. Neurosci.* 11 127–138. 10.1038/nrn2787 20068583

[B47] Friston K. J. (2011). Functional and effective connectivity: a review. *Brain Connect.* 1 13–36. 10.1089/brain.2011.0008 22432952

[B48] Friston K. J. (2013). Life as we know it. *J. R. Soc. Interf.* 10:20130475. 10.1098/rsif.2013.0475 23825119 PMC3730701

[B49] Friston K. J. (2019). A free energy principle for a particular physics. *ArXiv* [Preprint] ArXiv:1906.10184 [q-Bio], 10.1016/j.plrev.2022.05.002 35588546

[B50] Friston K. J. Brown H. R. Siemerkus J. Stephan K. E. (2016). The dysconnection hypothesis (2016). *Schizophr. Res.* 176 83–94. 10.1016/j.schres.2016.07.014 27450778 PMC5147460

[B51] Friston K. J. Fagerholm E. D. Zarghami T. S. Parr T. Hipólito I. Magrou L. (2021). Parcels and particles: Markov blankets in the brain. *Netw. Neurosci.* 5 211–251. 10.1162/netn_a_00175 33688613 PMC7935044

[B52] Friston K. J. Harrison L. Penny W. (2003). Dynamic causal modelling. *Neuroimage* 19 1273–1302. 10.1016/S1053-8119(03)00202-7 12948688

[B53] Friston K. J. Kahan J. Razi A. Stephan K. E. Sporns O. (2014). On nodes and modes in resting state fMRI. *Neuroimage* 99 533–547. 10.1016/j.neuroimage.2014.05.056 24862075 PMC4121089

[B54] Friston K. J. Parr T. de Vries B. (2017a). The graphical brain: belief propagation and active inference. *Netw. Neurosci.* 1 381–414. 10.1162/NETN_a_00018 29417960 PMC5798592

[B55] Friston K. J. Preller K. H. Mathys C. Cagnan H. Heinzle J. Razi A. (2019). Dynamic causal modelling revisited. *Neuroimage* 199 730–744. 10.1016/j.neuroimage.2017.02.045 28219774 PMC6693530

[B56] Friston K. J. Redish A. D. Gordon J. A. (2017b). Computational nosology and precision psychiatry. *Comput. Psychiatry* 1 2–23. 10.1162/CPSY_a_00001 PMC5774181 29400354

[B57] Friston K. J. Wiese W. Hobson J. A. (2020). Sentience and the origins of consciousness: from cartesian duality to Markovian monism. *Entropy* 22:516. 10.3390/e22050516 33286288 PMC7517007

[B58] Gentner D. (2010). Bootstrapping the mind: analogical processes and symbol systems. *Cogn. Sci.* 34 752–775. 10.1111/j.1551-6709.2010.01114.x 21564235

[B59] Gerraty R. T. Davidow J. Y. Foerde K. Galvan A. Bassett D. S. Shohamy D. (2018). Dynamic flexibility in striatal-cortical circuits supports reinforcement learning. *J. Neurosci.* 38 2442–2453. 10.1523/JNEUROSCI.2084-17.2018 29431652 PMC5858591

[B60] Girn M. Roseman L. Bernhardt B. Smallwood J. Carhart-Harris R. Spreng R. N. (2021). Serotonergic psychedelic drugs LSD and psilocybin reduce the hierarchical differentiation of unimodal and transmodal cortex. *bioRxiv* [Preprint] 10.1101/2020.05.01.072314 35483649

[B61] Gopnik A. O’Grady S. Lucas C. G. Griffiths T. L. Wente A. Bridgers S. (2017). Changes in cognitive flexibility and hypothesis search across human life history from childhood to adolescence to adulthood. *Proc. Natl. Acad. Sci. U.S.A.* 114 7892–7899. 10.1073/pnas.1700811114 28739917 PMC5544286

[B62] Haken H. (1983). *Synergetics: An Introduction?: Nonequilibrium Phase Transitions and Self-organization in Physics, Chemistry, and Biology.* Berlin: Springer.

[B63] Haken H. Kelso J. A. S. Bunz H. (1985). A theoretical model of phase transitions in human hand movements. *Biol. Cybern.* 51 347–356. 10.1007/BF00336922 3978150

[B64] Hanson A. (2021). Spontaneous electrical low-frequency oscillations: a possible role in *Hydra* and all living systems. *Philos. Trans. R. Soc. B Biol. Sci.* 376:20190763. 10.1098/rstb.2019.0763 33487108 PMC7934974

[B65] Hassabis D. Maguire E. A. (2009). The construction system of the brain. *Philos. Trans. R. Soc. Lond. Ser. B Biol. Sci.* 364 1263–1271. 10.1098/rstb.2008.0296 19528007 PMC2666702

[B66] Haueis P. (2021). Multiscale modeling of cortical gradients: the role of mesoscale circuits for linking macro- and microscale gradients of cortical organization and hierarchical information processing. *Neuroimage* 232:117846. 10.1016/j.neuroimage.2021.117846 33636345

[B67] Hayes S. C. (2019). *A Liberated Mind: How to Pivot Toward What Matters.* New York, NY: Penguin.

[B68] He L. Zhuang K. Li Y. Sun J. Meng J. Zhu W. (2019). Brain flexibility associated with need for cognition contributes to creative achievement. *Psychophysiology* 56:e13464. 10.1111/psyp.13464 31453642

[B69] Herzog R. Mediano P. A. M. Rosas F. E. Carhart-Harris R. Perl Y. S. Tagliazucchi E. (2020). A mechanistic model of the neural entropy increase elicited by psychedelic drugs. *Sci. Rep.* 10:17725. 10.1038/s41598-020-74060-6 33082424 PMC7575594

[B70] Heylighen F. (2016). Stigmergy as a universal coordination mechanism I: definition and components. *Cogn. Syst. Res.* 38 4–13. 10.1016/j.cogsys.2015.12.002

[B71] Hinton D. E. Kirmayer L. J. (2017). The flexibility hypothesis of healing. *Cult. Med. Psychiatry* 41 3–34. 10.1007/s11013-016-9493-8 27142641

[B72] Hipólito I. Baltieri M. Friston K. Ramstead M. J. D. (2021a). Embodied skillful performance: where the action is. *Synthese* 199 4457–4481. 10.1007/s11229-020-02986-5 34866668 PMC8602225

[B73] Hipólito I. Ramstead M. J. D. Convertino L. Bhat A. Friston K. Parr T. (2021b). Markov blankets in the brain. *Neurosci. Biobehav. Rev.* 125 88–97. 10.1016/j.neubiorev.2021.02.003 33607182 PMC8373616

[B74] Hoffmann H. Payton D. W. (2018). Optimization by self-organized criticality. *Sci. Rep.* 8:2358. 10.1038/s41598-018-20275-7 29402956 PMC5799203

[B75] Hofstadter D. Sander E. (2013). *Surfaces and Essences: Analogy as the Fuel and Fire of Thinking*, 1 Edn. New York, NY: Basic Books.

[B76] Huang Z. Zhang J. Wu J. Mashour G. A. Hudetz A. G. (2020). Temporal circuit of macroscale dynamic brain activity supports human consciousness. *Sci. Adv.* 6:eaaz0087. 10.1126/sciadv.aaz0087 32195349 PMC7065875

[B77] Jaeger J. Monk N. (2021). Dynamical modules in metabolism, cell and developmental biology. *OSF Preprints* 10.31219/osf.io/rydbn PMC8086940 34055307

[B78] Jafarian A. Zeidman P. Wykes R. C. Walker M. Friston K. J. (2021). Adiabatic dynamic causal modelling. *Neuroimage* 238:118243. 10.1016/j.neuroimage.2021.118243 34116151 PMC8350149

[B79] Jirsa V. K. Friedrich R. Haken H. Kelso J. A. S. (1994). A theoretical model of phase transitions in the human brain. *Biol. Cybern.* 71 27–35. 10.1007/BF00198909 8054384

[B80] Jutla I. S. Jeub L. G. Mucha P. J. (2011). *A Generalized Louvain Method for Community Detection Implemented in MATLAB.* Available online at: http://Netwiki.Amath.Unc.Edu/GenLouvain (accessed December 1, 2021).

[B81] Kenett Y. N. Medaglia J. D. Beaty R. E. Chen Q. Betzel R. F. Thompson-Schill S. L. (2018). Driving the brain towards creativity and intelligence: a network control theory analysis. *Neuropsychologia* 118 79–90. 10.1016/j.neuropsychologia.2018.01.001 29307585 PMC6034981

[B82] Kirchhoff M. Parr T. Palacios E. Friston K. J. Kiverstein J. (2018). The Markov blankets of life: autonomy, active inference and the free energy principle. *J. R. Soc. Interf.* 15:20170792. 10.1098/rsif.2017.0792 29343629 PMC5805980

[B83] Koch C. (1999). *Biophysics of Computation: Information Processing in Single Neurons.* New York, NY: Oxford University Press.

[B84] Kugler P. N. Scott Kelso J. A. Turvey M. T. (1980). “1 On the concept of coordinative structures as dissipative structures: I. Theoretical lines of convergence**this work was supported by NIH grants HD 01994, NS 13617 and AM 25814,” in *Advances in Psychology*, Vol. 1 eds Stelmach G. E. Requin J. (Amsterdam: North-Holland), 3–47. 10.1016/S0166-4115(08)61936-6

[B85] Lahav N. Sendiña-Nadal I. Hens C. Ksherim B. Barzel B. Cohen R. (2018). Synchronization of chaotic systems: a microscopic description. *Phys. Rev. E* 98:052204. 10.1103/PhysRevE.98.052204 PMC8847423 35169176

[B86] Lamprecht I. Zotin A. I. (2019). *Thermodynamics of Biological Processes.* Berlin: De Gruyter. 10.1515/9783110860511

[B87] Lee C. Wilkinson D. J. (2019). A review of stochastic block models and extensions for graph clustering. *Appl. Netw. Sci.* 4 1–50. 10.1007/s41109-019-0232-2

[B88] Li R. Ryu J. H. Vincent P. Springer M. Kluger D. Levinsohn E. A. (2021). The pulse: transient fMRI signal increases in subcortical arousal systems during transitions in attention. *Neuroimage* 232:117873. 10.1016/j.neuroimage.2021.117873 33647499 PMC8278331

[B89] Lyapunov A. M. (1992). The general problem of the stability of motion. *Int. J. Control* 55 531–534. 10.1080/00207179208934253

[B90] Mashour G. A. Roelfsema P. Changeux J.-P. Dehaene S. (2020). Conscious processing and the global neuronal workspace hypothesis. *Neuron* 105 776–798. 10.1016/j.neuron.2020.01.026 32135090 PMC8770991

[B91] Massimini M. Ferrarelli F. Sarasso S. Tononi G. (2012). Cortical mechanisms of loss of consciousness: insight from TMS/EEG studies. *Arch. Ital. Biol.* 150 44–55. 23165870 10.4449/aib.v150i2.1361

[B92] Mattar M. Bassett D. (2019). “Brain network architecture: implications for human learning,” in *Network Science in Cognitive Psychology*, ed. Vitevitch M. S. (Milton Park: Routledge), 30–44. 10.4324/9780367853259-3

[B93] Mayner W. G. P. Marshall W. Albantakis L. Findlay G. Marchman R. Tononi G. (2018). PyPhi: a toolbox for integrated information theory. *PLoS Comput. Biol.* 14:e1006343. 10.1371/journal.pcbi.1006343 30048445 PMC6080800

[B94] Mediano P. A. M. Seth A. K. Barrett A. B. (2019). Measuring integrated information: comparison of candidate measures in theory and simulation. *Entropy* 21:17. 10.3390/e21010017 33266733 PMC7514120

[B95] Michel C. M. Koenig T. (2018). EEG microstates as a tool for studying the temporal dynamics of whole-brain neuronal networks: a review. *Neuroimage* 180 577–593. 10.1016/j.neuroimage.2017.11.062 29196270

[B96] Mišić B. Betzel R. F. Nematzadeh A. Goñi J. Griffa A. Hagmann P. (2015). Cooperative and competitive spreading dynamics on the human connectome. *Neuron* 86 1518–1529. 10.1016/j.neuron.2015.05.035 26087168

[B97] Northoff G. Tumati S. (2019). “Average is good, extremes are bad”—Non-linear inverted U-shaped relationship between neural mechanisms and functionality of mental features. *Neurosci. Biobehav. Rev.* 104 11–25. 10.1016/j.neubiorev.2019.06.030 31251964

[B98] Oligschläger S. Xu T. Baczkowski B. M. Falkiewicz M. Falchier A. Linn G. (2019). Gradients of connectivity distance in the cerebral cortex of the macaque monkey. *Brain Struct. Funct.* 224 925–935. 10.1007/s00429-018-1811-1 30547311 PMC6420469

[B99] Oliveira H. M. Melo L. V. (2015). Huygens synchronization of two clocks. *Sci. Rep.* 5:11548. 10.1038/srep11548 26204557 PMC4512151

[B100] Palacios E. R. Razi A. Parr T. Kirchhoff M. Friston K. (2017). Biological self-organisation and Markov blankets. *bioRxiv* [Preprint] 10.1101/227181 PMC7284313 31756340

[B101] Paperin G. Green D. G. Sadedin S. (2011). Dual-phase evolution in complex adaptive systems. *J. R. Soc. Interf.* 8 609–629. 10.1098/rsif.2010.0719 21247947 PMC3061102

[B102] Parr T. Da Costa L. Friston K. (2020). Markov blankets, information geometry and stochastic thermodynamics. *Philos. Trans. R. Soc. A Math. Phys. Eng. Sci.* 378:20190159. 10.1098/rsta.2019.0159 31865883 PMC6939234

[B103] Parr T. Friston K. J. (2018). The anatomy of inference: generative models and brain structure. *Front. Comput. Neurosci.* 12:90. 10.3389/fncom.2018.00090 30483088 PMC6243103

[B104] Parr T. Limanowski J. Rawji V. Friston K. (2021). The computational neurology of movement under active inference. *Brain* 144 1799–1818. 10.1093/brain/awab085 33704439 PMC8320263

[B105] Pavlos G. P. Karakatsanis L. P. Xenakis M. N. Sarafopoulos D. Pavlos E. G. (2012). Tsallis statistics and magnetospheric self-organization. *Physica A* 391 3069–3080. 10.1016/j.physa.2012.01.033

[B106] Pedersen M. Zalesky A. Omidvarnia A. Jackson G. D. (2018). Multilayer network switching rate predicts brain performance. *Proc. Natl. Acad. Sci. U.S.A.* 115 13376–13381. 10.1073/pnas.1814785115 30545918 PMC6310789

[B107] Penn D. C. Holyoak K. J. Povinelli D. J. (2008). Darwin’s mistake: explaining the discontinuity between human and nonhuman minds. *Behav. Brain Sci.* 31 109–130; discussion 130–178. 10.1017/S0140525X08003543 18479531

[B108] Poldrack R. A. (2020). The physics of representation. *Synthese* 199 1307–1325. 10.1007/s11229-020-02793-y

[B109] Rabinovich M. Huerta R. Laurent G. (2008). Transient dynamics for neural processing. *Science* 321 48–50. 18599763 10.1126/science.1155564

[B110] Rabinovich M. I. Afraimovich V. S. Bick C. Varona P. (2012). Information flow dynamics in the brain. *Phys. Life Rev.* 9 51–73. 10.1016/j.plrev.2011.11.002 22119154

[B111] Rabinovich M. I. Varona P. (2018). Discrete sequential information coding: heteroclinic cognitive dynamics. *Front. Comput. Neurosci.* 12:73. 10.3389/fncom.2018.00073 30245621 PMC6137616

[B112] Ramstead M. J. D. Friston K. J. Hipólito I. (2020). Is the free-energy principle a formal theory of semantics? From variational density dynamics to neural and phenotypic representations. *Entropy* 22:889. 10.3390/e22080889 33286659 PMC7517505

[B113] Razi A. Friston K. J. (2016). The connected brain: causality, models, and intrinsic dynamics. *IEEE Signal Process. Mag.* 33 14–35. 10.1109/MSP.2015.2482121

[B114] Rosvall M. Bergstrom C. T. (2008). Maps of random walks on complex networks reveal community structure. *Proc. Natl. Acad. Sci. U.S.A.* 105 1118–1123. 10.1073/pnas.0706851105 18216267 PMC2234100

[B115] Roweis S. Ghahramani Z. (1999). A unifying review of linear gaussian models. *Neural Comput.* 11 305–345. 10.1162/089976699300016674 9950734

[B116] Rueter A. R. Abram S. V. MacDonald A. W. Rustichini A. DeYoung C. G. (2018). The goal priority network as a neural substrate of conscientiousness. *Hum. Brain Mapp.* 39 3574–3585. 10.1002/hbm.24195 29691946 PMC6200659

[B117] Safron A. (2020). An integrated world modeling theory (IWMT) of consciousness: combining integrated information and global neuronal workspace theories with the free energy principle and active inference framework; toward solving the hard problem and characterizing agentic causation. *Front. Artif. Intell.* 3:30. 10.3389/frai.2020.00030 33733149 PMC7861340

[B118] Safron A. (2021). Integrated world modeling theory (IWMT) expanded: implications for theories of consciousness and artificial intelligence. *PsyArXiv* [Preprint] 10.31234/osf.io/rm5b2

[B119] Safron A. Çatal O. Verbelen T. (2021). Generalized simultaneous localization and mapping (G-SLAM) as unification framework for natural and artificial intelligences: towards reverse engineering the hippocampal/entorhinal system and principles of high-level cognition. *PsyArXiv* [Preprint] 10.31234/osf.io/tdw82 PMC9563348 36246500

[B120] Safron A. DeYoung C. G. (2021). “Chapter 18 - Integrating cybernetic big five theory with the free energy principle: a new strategy for modeling personalities as complex systems,” in *Measuring and Modeling Persons and Situations*, eds Wood D. Read S. J. Harms P. D. Slaughter A. (Cambridge, MA: Academic Press), 617–649. 10.1016/B978-0-12-819200-9.00010-7

[B121] Saxe R. Moran J. M. Scholz J. Gabrieli J. (2006). Overlapping and non-overlapping brain regions for theory of mind and self reflection in individual subjects. *Soc. Cogn. Affect. Neurosci.* 1 229–234. 10.1093/scan/nsl034 18985110 PMC2555418

[B122] Shanahan M. (2012). The brain’s connective core and its role in animal cognition. *Philos. Trans. R. Soc. B Biol. Sci.* 367 2704–2714. 10.1098/rstb.2012.0128 22927569 PMC3427545

[B123] Shine J. M. (2019). Neuromodulatory influences on integration and segregation in the brain. *Trends Cogn. Sci.* 23 572–583. 10.1016/j.tics.2019.04.002 31076192

[B124] Shine J. M. (2021). The thalamus integrates the macrosystems of the brain to facilitate complex, adaptive brain network dynamics. *Prog. Neurobiol.* 199:101951. 10.1016/j.pneurobio.2020.101951 33189781

[B125] Shine J. M. Breakspear M. Bell P. Martens K. E. Shine R. Koyejo O. (2018). The dynamic basis of cognition: an integrative core under the control of the ascending neuromodulatory system. *BioRxiv* [Preprint] 10.1101/266635

[B126] Sizemore A. E. Bassett D. S. (2018). Dynamic graph metrics: tutorial, toolbox, and tale. *Neuroimage* 180 417–427. 10.1016/j.neuroimage.2017.06.081 28698107 PMC5758445

[B127] Smith L. Byrge L. Sporns O. (2020). “Beyond origins: developmental pathways and the dynamics of brain networks,” in *Current Controversies in Philosophy of Cognitive Science*, eds Lerner A. J. Cullen S. Leslie S. J. (New York, NY: Routledge), 49–62. 10.4324/9781003026273-7

[B128] Sneppen K. Bak P. Flyvbjerg H. Jensen M. H. (1995). Evolution as a self-organized critical phenomenon. *Proc. Natl. Acad. Sci. U.S.A.* 92 5209–5213. 7761475 10.1073/pnas.92.11.5209 PMC41878

[B129] Sporns O. (2013). Network attributes for segregation and integration in the human brain. *Curr. Opin. Neurobiol.* 23 162–171. 10.1016/j.conb.2012.11.015 23294553

[B130] Sporns O. Betzel R. F. (2016). Modular brain networks. *Annu. Rev. Psychol.* 67 613–640. 10.1146/annurev-psych-122414-033634 26393868 PMC4782188

[B131] Standage D. Areshenkoff C. N. Nashed J. Y. Hutchison R. M. Hutchison M. Heinke D. (2020). Dynamic reconfiguration, fragmentation, and integration of whole-brain modular structure across depths of unconsciousness. *Cereb. Cortex* 30 5229–5241. 10.1093/cercor/bhaa085 32469053 PMC7472202

[B132] Stone E. Holmes P. (1990). Random perturbations of heteroclinic attractors. *SIAM J. Appl. Math.* 50 726–743.

[B133] Tegmark M. (2016). Improved measures of integrated information. *PLoS Comput. Biol.* 12:e1005123. 10.1371/journal.pcbi.1005123 27870846 PMC5117999

[B134] Telesford Q. K. Ashourvan A. Wymbs N. F. Grafton S. T. Vettel J. M. Bassett D. S. (2017). Cohesive network reconfiguration accompanies extended training. *Hum. Brain Mapp.* 38 4744–4759. 10.1002/hbm.23699 28646563 PMC5554863

[B135] Tononi G. Boly M. Massimini M. Koch C. (2016). Integrated information theory: from consciousness to its physical substrate. *Nat. Rev. Neurosci.* 17:450. 10.1038/nrn.2016.44 27225071

[B136] Tononi G. Edelman G. (1998). Consciousness and complexity. *Science* 282 1846–1851. 9836628 10.1126/science.282.5395.1846

[B137] Toschi N. Riccelli R. Indovina I. Terracciano A. Passamonti L. (2018). Functional connectome of the five-factor model of personality. *Pers. Neurosci.* 1:e2. 10.1017/pen.2017.2 30294715 PMC6171528

[B138] Touboul J. Destexhe A. (2017). Power-law statistics and universal scaling in the absence of criticality. *Phys. Rev. E* 95:012413. 10.1103/PhysRevE.95.012413 28208383

[B139] Uddin L. Q. (2021). Cognitive and behavioural flexibility: neural mechanisms and clinical considerations. *Nat. Rev. Neurosci.* 22 167–179. 10.1038/s41583-021-00428-w 33536614 PMC7856857

[B140] Utevsky A. V. Smith D. V. Huettel S. A. (2014). Precuneus is a functional core of the default-mode network. *J. Neurosci.* 34 932–940. 10.1523/JNEUROSCI.4227-13.2014 24431451 PMC3891968

[B141] van den Heuvel M. P. Kahn R. S. Goñi J. Sporns O. (2012). High-cost, high-capacity backbone for global brain communication. *Proc. Natl. Acad. Sci. U.S.A.* 109 11372–11377. 10.1073/pnas.1203593109 22711833 PMC3396547

[B142] van den Heuvel M. P. Scholtens L. H. de Lange S. C. Pijnenburg R. Cahn W. van Haren N. E. M. (2019). Evolutionary modifications in human brain connectivity associated with schizophrenia. *Brain J. Neurol.* 142 3991–4002. 10.1093/brain/awz330 31724729 PMC6906591

[B143] van Es T. Hipolito I. (2020). *Free-Energy Principle, Computationalism and Realism: A Tragedy [Preprint].* Available online at: http://philsci-archive.pitt.edu/18497/ (accessed December 7, 2020)

[B144] Van Gelder T. (1995). What might cognition be, if not computation? *J. Philos.* 92 345–381. 10.2307/2941061

[B145] Varley T. Craig M. Adapa R. Finoia P. Williams G. Allanson J. (2019). Fractal dimension of cortical functional connectivity networks predicts severity in disorders of consciousness. *BioRxiv* [Preprint] BioRxiv:789636, 10.1371/journal.pone.0223812 PMC7017993 32053587

[B146] Váša F. Shanahan M. Hellyer P. J. Scott G. Cabral J. Leech R. (2015). Effects of lesions on synchrony and metastability in cortical networks. *Neuroimage* 118 456–467. 10.1016/j.neuroimage.2015.05.042 26049146

[B147] Vázquez-Rodríguez B. Avena-Koenigsberger A. Sporns O. Griffa A. Hagmann P. Larralde H. (2017). Stochastic resonance at criticality in a network model of the human cortex. *Sci. Rep.* 7:13020. 10.1038/s41598-017-13400-5 29026142 PMC5638949

[B148] Vázquez-Rodríguez B. Suárez L. E. Markello R. D. Shafiei G. Paquola C. Hagmann P. (2019). Gradients of structure–function tethering across neocortex. *Proc. Natl. Acad. Sci. U.S.A.* 116 21219–21227. 10.1073/pnas.1903403116 31570622 PMC6800358

[B149] Vidaurre D. Smith S. M. Woolrich M. W. (2017). Brain network dynamics are hierarchically organized in time. *Proc. Natl. Acad. Sci. U.S.A.* 114 12827–12832. 10.1073/pnas.1705120114 29087305 PMC5715736

[B150] Vivot R. M. Pallavicini C. Zamberlan F. Vigo D. Tagliazucchi E. (2020). Meditation increases the entropy of brain oscillatory activity. *Neuroscience* 431 40–51. 10.1016/j.neuroscience.2020.01.033 32032666

[B151] Walsh K. S. McGovern D. P. Clark A. O’Connell R. G. (2020). Evaluating the neurophysiological evidence for predictive processing as a model of perception. *Ann. N. Y. Acad. Sci.* 1464 242–268. 10.1111/nyas.14321 32147856 PMC7187369

[B152] Wens V. Bourguignon M. Vander Ghinst M. Mary A. Marty B. Coquelet N. (2019). Synchrony, metastability, dynamic integration, and competition in the spontaneous functional connectivity of the human brain. *Neuroimage* 199 313–324. 10.1016/j.neuroimage.2019.05.081 31170458

[B153] Yan K. Hricko J. (2017). Brain networks, structural realism, and local approaches to the scientific realism debate. *Stud. Hist. Philos. Sci. C Stud. Hist. Philos. Biol. Biomed. Sci.* 64 1–10. 10.1016/j.shpsc.2017.05.001 28499176

[B154] Ye Q. Zou F. Lau H. Hu Y. Kwok S. C. (2018). Causal evidence for mnemonic metacognition in human precuneus. *J. Neurosci.* 38 6379–6387. 10.1523/JNEUROSCI.0660-18.2018 29921714 PMC6041789

[B155] Yerkes R. M. Dodson J. D. (1908). The relation of strength of stimulus to rapidity of habit-formation. *J. Comp. Neurol. Psychol.* 18 459–482. 10.1002/cne.920180503

[B156] Yin W. Li T. Hung S.-C. Zhang H. Wang L. Shen D. (2020). The emergence of a functionally flexible brain during early infancy. *Proc. Natl. Acad. Sci. U.S.A.* 117 23904–23913. 10.1073/pnas.2002645117 32868436 PMC7519318

[B157] Yuan R. Ma Y. Yuan B. Ao P. (2011). “Potential function in dynamical systems and the relation with Lyapunov function,” in *Proceedings of the 30th Chinese Control Conference*, Yantai, 6573–6580.

[B158] Zarghami T. S. Friston K. J. (2020). Dynamic effective connectivity. *Neuroimage* 207:116453. 10.1016/j.neuroimage.2019.116453 31821868

